# Phlebotomine sand flies (Diptera, Psychodidae) of the world

**DOI:** 10.1186/s13071-025-06748-5

**Published:** 2025-06-10

**Authors:** Eunice Aparecida Bianchi Galati, Andrey José de Andrade, Farzana Perveen, Mathieu Loyer, Khamsing Vongphayloth, Fano José Randrianambinintsoa, Jorian Prudhomme, Nil Rahola, Mohammad Akhoundi, Paloma Helena Fernandes Shimabukuro, Jérôme Depaquit

**Affiliations:** 1 Faculdade de Saúde Pública da Universidade de São Paulo (FSP/USP), Senior Professor Program, Av. Dr. Arnaldo, 715, São Paulo, Brazil; 2 Coleção de Parasitologia do Departamento de Patologia Básica (ColPar/Dpat), Setor de Ciências Biológicas, Universidade Federal do Paraná, Curitiba, Paraná Brazil; 3 Faculté de Pharmacie, Université de Reims Champagne Ardenne, UR ESCAPE-USC ANSES PETARD, 51 rue Cognacq-Jay, 51096 Reims Cedex, France; 4 Laboratory of Vector-Borne Diseases, Institut Pasteur du Laos, Samsenhai Road, Ban Kao-Gnot, Sisattanak District, 3560 Vientiane, Lao PDR; 5 MIVEGEC, Univ. Montpellier, CNRS, IRD, Montpellier, France; 6 Medical Entomology Unit, Institut Pasteur de Madagascar, Antananarivo, Madagascar; 7 Parasitology-Mycology Department, Avicenne Hospital, AP-HP, Bobigny, Sorbonne Paris Nord University, Villetaneuse, France; 8 Unité des Virus Émergents (UVE: Aix-Marseille Université, Università di Corsica, IRD 190-Inserm 1207-IHU Méditerranée Infection), Marseille, France; 9 Grupo de Estudos em Leishmanioses/Coleção de Flebotomíneos (COLFLEB/Fiocruz-MG), Instituto René Rachou, Fundação Oswaldo Cruz, Belo Horizonte, Minas Gerais Brazil; 10 pôle de Biologie territoriale, Laboratoire de Parasitologie-Mycologie, Centre Hospitalo-Universitaire, 51092 Reims, France

**Keywords:** Geographical distribution, Sand flies, New World, Nomenclature changes, Old World, Taxonomy, Systematics, World fauna

## Abstract

**Background:**

Checklists of zoological groups are useful to document species names in a specific area or even worldwide. They serve for various purposes, including ecological studies, conservation reports, policy and decision-making, and species identification. Phlebotomine sand flies (Diptera, Psychodidae) are the vectors of pathogens such as *Leishmania*, *Bartonella* and some arboviruses (Toscana, Naples, Sicily), and checklists for sand flies have primarily been published mainly for a state, department, or country. A checklist for American sand flies was published in 2017, but, until then, no effort has been made to compile a comprehensive list of species worldwide. The present study aims to fill this gap of knowledge.

**Methods:**

The present checklist is provided based on a literature overview and biological collections records and includes unpublished data from the authors. The species are presented according to the classification, then alphabetically by Eastern and Western Hemispheres. Distribution by country and type locality of each species are provided. Discussions on the taxonomic status or occurrence of each species are provided when needed.

**Results:**

A total of 23 genera in the Western Hemisphere, formerly the New World (*Bichromomyia*, *Brumptomyia*, *Deanemyia*, *Dampfomyia, Edentomyia, Evandromyia, Expapillata, Hertigia, Lutzomyia, Martinsmyia, Micropygomyia, Migonemyia, Nyssomyia, Oligodontomyia, Pintomyia, Pressatia, Psathyromyia, Psychodopygus, Sciopemyia, Trichophoromyia, Trichopygomyia, Viannamyia*, and *Warileya*) and 17 genera in the Eastern Hemisphere, formerly the Old World (*Australophlebotomus, Chinius, Demeillonius, Grassomyia, Idiophlebotomus, Libanophlebotomus, Libanophlebotomites, Mesophlebotomites, Paleomyia, Parvidens, Phlebotoiella, Phlebotomiella, Phlebotomites, Phlebotomus, Sergentomyia, Spelaeomyia, Spelaeophlebotomus*), including the fossil species, are listed herein. The updated list, excluding *nomina dubia*/*species inquirenda* includes 1063 sand fly species, with 549 and 514 species for New World and Old World, respectively. Only New Zealand and the Pacific Islands (excluding New Caledonia) do not record the presence of sand flies. The dataset for this study is publicly available in the SiBBr and GBIF.

**Conclusions:**

This is the first detailed list of valid names of phlebotomine sand flies worldwide, including records from each country where they have been documented.

**Graphical Abstract:**

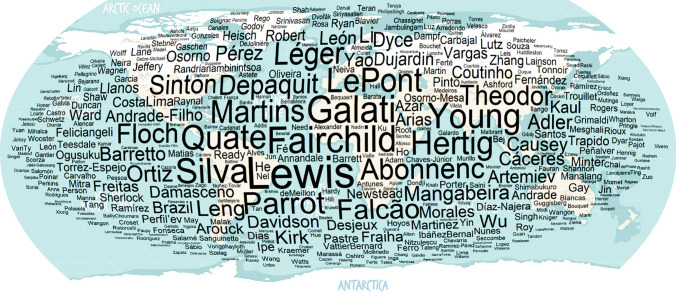

## Background

Phlebotomine sand flies (Diptera, Psychodidae) are medically important insects that include vectors of pathogens such as *Leishmania*, *Bartonella*, and some arboviruses [1]. Recently, Galati and Rodrigues [2] published an extensive global review of the Phlebotominae subfamily documenting 1060 taxa of the group of species (species and subspecies) across the Western and Eastern Hemispheres. A timeline of the Phlebotominae systematics was presented, highlighting the type localities, the number of authors involved in species descriptions, and the contributions of researchers and their respective institutions. A table summarizing the current number of species at each respective taxonomic level and their geographical distribution by hemisphere was also compiled.

For New World sand flies, the classification proposed by Galati addressed some key taxonomic questions on the relationships of American species [3–6]. This classification has been supported for several supraspecífic taxa by several authors using molecular markers [7–11]. Conversely, the systematics of Old World sand flies requires a thorough taxonomic review [12, 13], especially for the 329 valid species names, as well as six names that are invalid under the provisions of the International Code of Zoological Nomenclature (ICZN) [14] within the genus *Sergentomyia* França and Parrot, 1920, including studies of intra and interspecific variations. Léger et al. [12] also raised questions about the validity of the subgenus *Neophlebotomus* França and Parrot, 1920 [15], which was proposed to group the sand fly species close to *Phlebotomus* Rondani and Berté, 1840 and *Sergentomyia*. Lewis probably misunderstood the *Neophlebotomus* subgenus and considered *Rondanomyia* Theodor, 1958, as a junior synonym of *Neophlebotomus* [16], but according to Léger et al. [12], this synonymy was not well supported leading to its revalidation. This example reinforces the urgent need for systematics and phylogeny studies for “Old World” sand flies [17].

Some authors published the distribution of sand flies by country or region [18–24]. The sand fly fauna is very diverse and widely distributed, with descriptions of new species being published annually [25–28]. Only New Zealand or the Pacific Islands, except New Caledonia, do not present records of sand flies [29].

## Methods

This checklist presents genera arranged according to the classification of Galati [5] for the “New World” and Lewis [16, 30, 31], Seccombe et al. [32], and Rispail and Léger [33, 34] for the “Old World”. Abbreviations of genus and subgenus follow Marcondes [35]. Subgenera, species groups/series, and species are listed alphabetically within each genus. Names that are considered *nomina dubia/species inquirenda* and names that denote more than one taxon according to provisions of the International Code of Zoological Nomenclature [14], articles 17.2 and 23.8, and unavailable names not meeting the requirements of the ICZN were excluded from this checklist following Shimabukuro et al. [20]. *Incertae sedis* refers to species for which the tribe, genus, subgenus, or species series have not been defined. Countries are listed alphabetically, and the country of the type locality is underlined. Fossil species are indicated by the symbol †. The species distribution is based on main papers and reviews of Lewis [16, 30, 31], Martins et al. [36], Seccombe et al. [32], Young and Duncan [37], Rispail and Léger [33, 34], Leng and Zhang [38], Niang et al. [39], Rueda et al. [40], Shimabukuro et al. [20], Galati [5, 41], Depaquit et al. [42], Vu et al. [23], Prudhomme et al. [43]. The authors of the present study evaluated the distribution area species by species. 

The complete list of species was compiled in an Excel^TM^ spreadsheet, formatted, and copied into a Darwin Core standard template [44] generated by the Nansen Legacy template generator [45]. Countries were georeferenced with Geonames (http://geonames.org/), and the centroid was used for all countries and Brazilian states, except for the USA and Russia where coordinates closer to the type locality were used. The dataset has undergone screening in the FIOCRUZ IPT (Integrated Publishing Toolkit), which is the Global Biodiversity Facility Information (GBIF) software to provide data through their network. Metadata fields are also available on the online pages and are publicly available in the “Sistema de Informação sobre a Biodiversidade Brasileira (SiBBr)” and the GBIF (10.15468/54hqx7) under a CC-BY license.

The figures were made using the packages tidyverse, ggwordcloud in R package.

This is the first effort in compiling the geographical distribution of sand flies by country by country in both New and Old Worlds. Worldwide, there are 33 extant genera, with 23 and 10 genera in the Western Hemisphere (formerly New World) and in the Eastern Hemisphere (formerly Old World), respectively, and seven genera exclusively fossil to the “Old World.” A total of 23 genera in the “New World” and 17 genera in the “Old World,” including the fossil species, are listed herein. The updated list includes 1057 sand fly taxa (species and subspecies), 549 and 514 for New World and Old World, respectively. Number of species by genus was pointed.

Summary statistics such as those for genera, species, and type locality country indication can be found in GBIF (https://www.gbif.org/dataset/193638ea-0d3c-4c23-865c-df53941174b8/metrics).

**Fig. 1:**
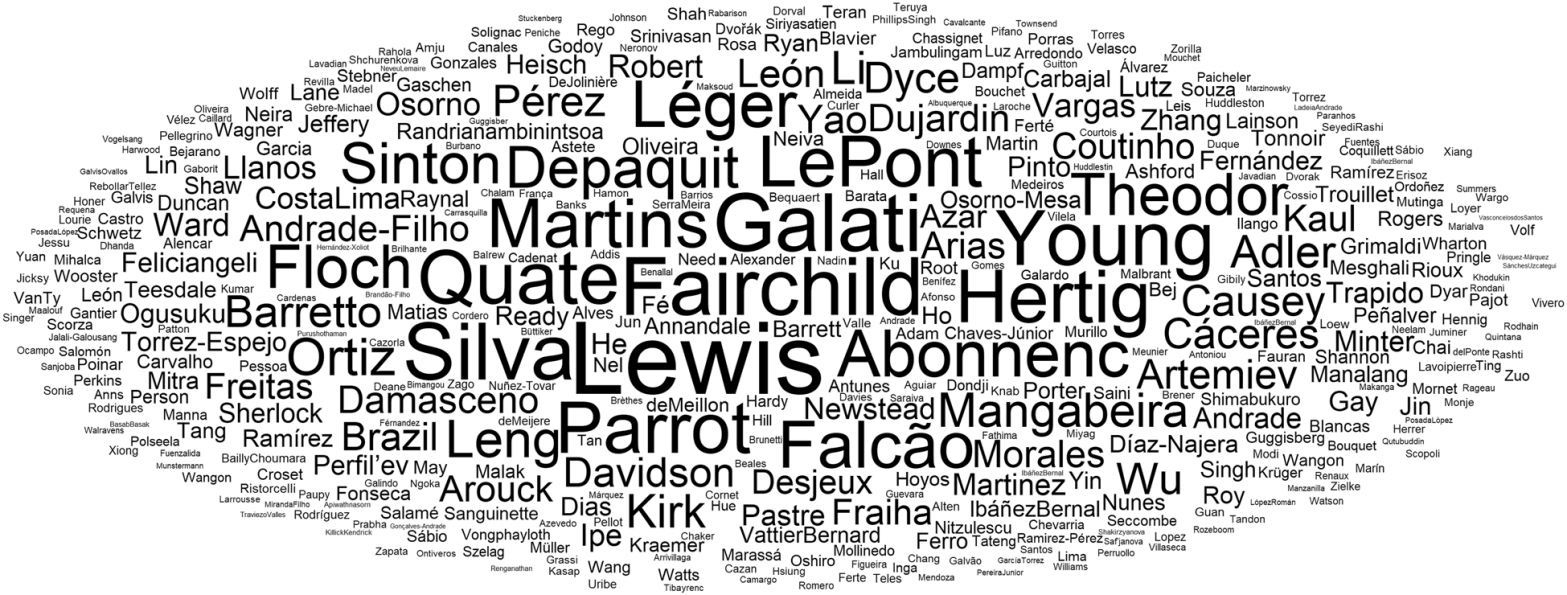
Word cloud with names of all authors of sand fly species. The size of words is equivalent to their frequency, larger words show authors who have described more species

**Fig. 2:**
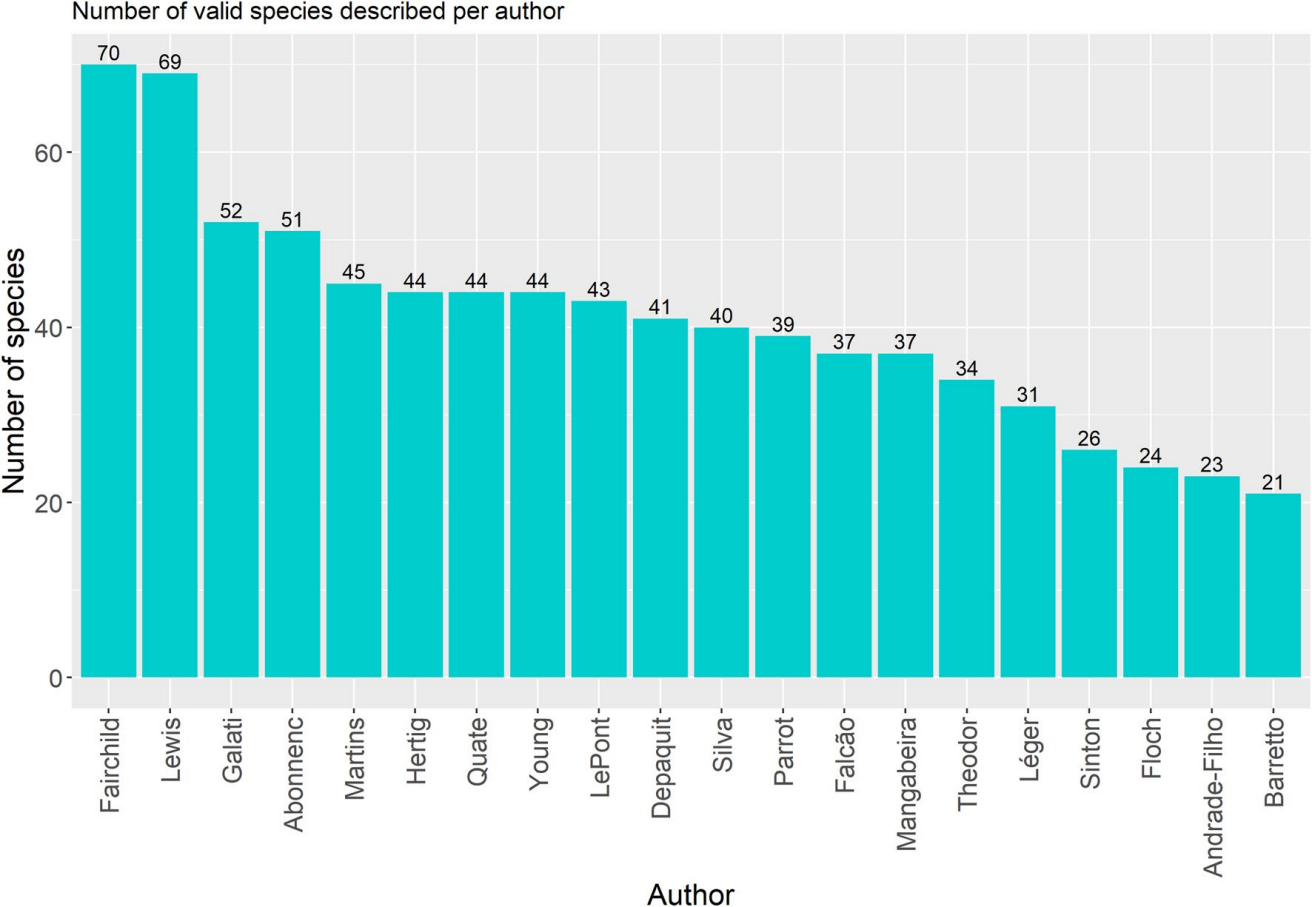
Authors responsible for the greater number of sand fly species described

**Fig. 3:**
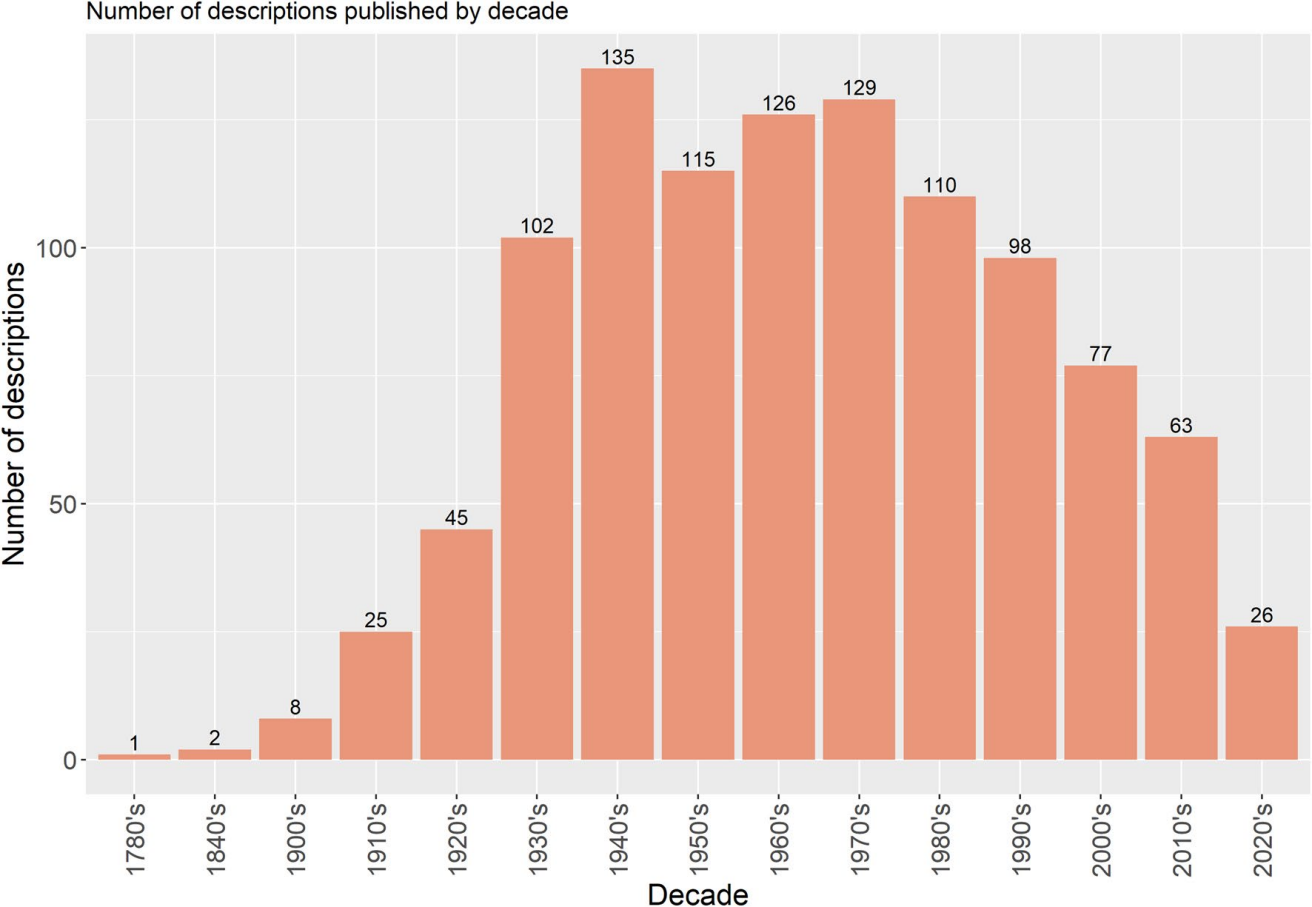
Number of sand flies described around the world per decade

**Fig. 4:**
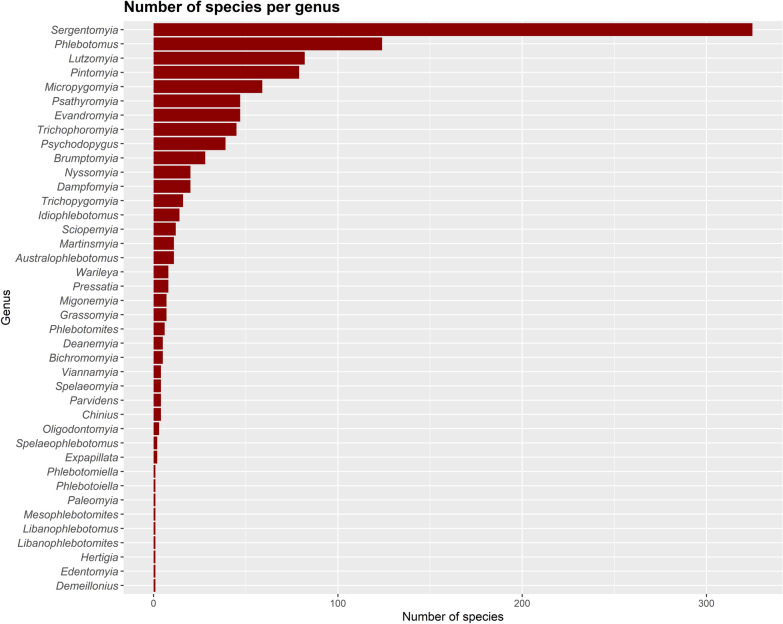
Number of world sand fly species by genera

**Fig. 5:**
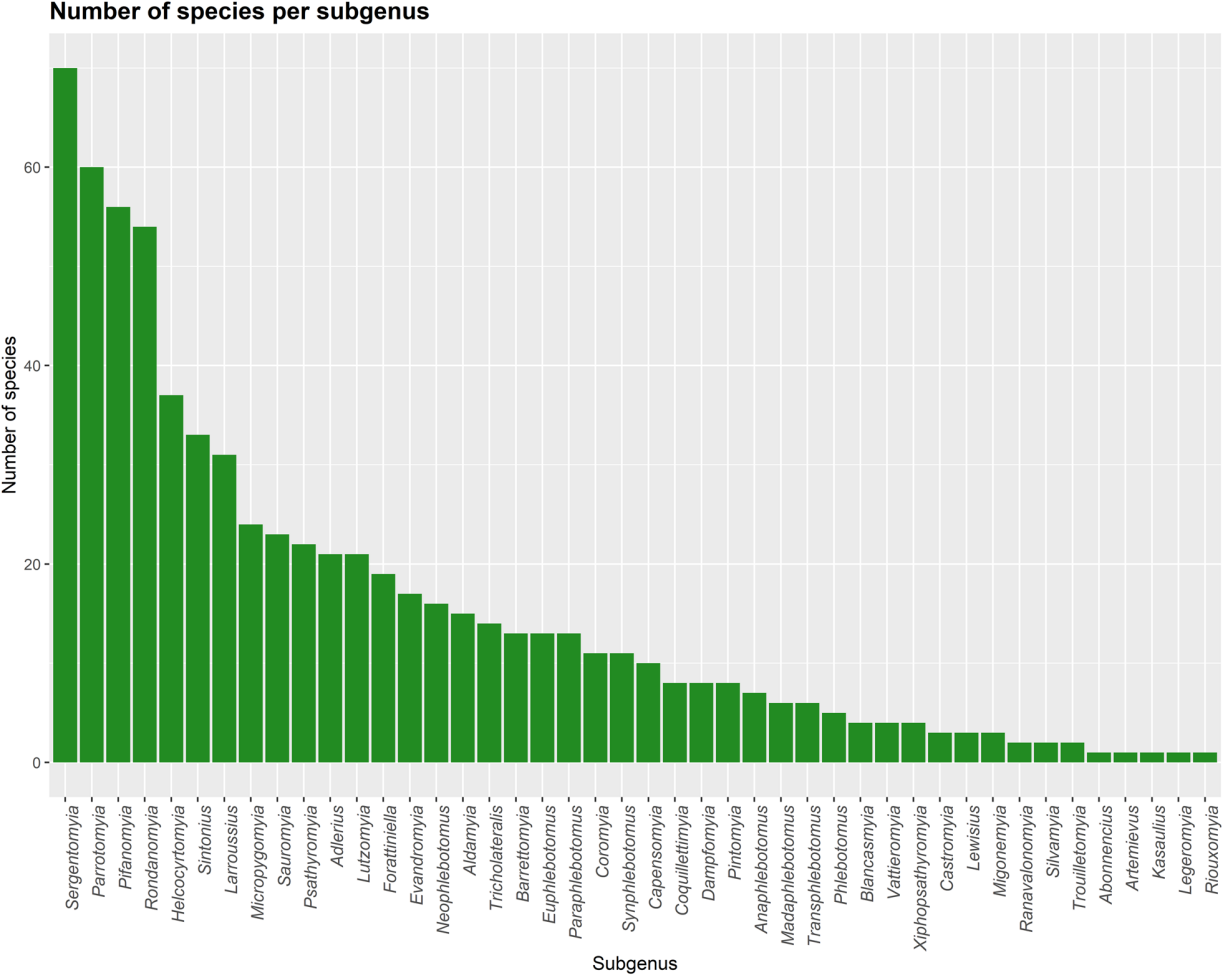
Number of sand fly species by subgenus or ungrouped categories in the world. Ungrouped taxa comprise one *Phlebotomus* fossil species and 76 species placed in the *Sergentomyia* genus

Worldwide, several authors contributed to the taxonomy of sand flies (Figs. 1 and 2), mainly after 30 decades (Fig. 3), when it was already well established that sand flies were vectors for *Leishmania*. The genera *Sergentomyia* and *Phlebotomus* are the most diverse, both occurring in the “Old World” (Fig. 4) and, excluding the ungrouped species, the subgenus *Pifanomyia* and *Riouxomyia* have the largest and smallest number of species (Fig. 5).

**Systematics**

Phylum Arthropoda von Siebold, 1848.

Subphylum Hexapoda Latreille, 1825.

Class Insecta Linnaeus, 1758.

Order Diptera Linnaeus, 1758.

Suborder Psychodomorpha Hennig, 1968.

Family Psychodidae Newman, 1834.

Subfamily Phlebotominae Rondani and Berté, 1840, in Rondani, 1840.

**Western Hemisphere (New World) (Total: 549 valid species)**

Tribe HERTIGIINI Abonnenc and Léger, 1976.

Subtribe HERTIGIINA Abonnenc and Léger, 1976.

*Hertigia* Fairchild, 1949 (1 species).

*Hertigia hertigi* Fairchild, 1949.

Distribution: Colombia, Costa Rica, Panama.

*Warileya* Hertig, 1948 (8 species).

*Warileya euniceae* Fernández, Carbajal, Astete and Wooster, 1998.

Distribution: Peru.

*Warileya fourgassiensis* Le Pont and Desjeux, 1984.

Distribution: French Guiana.

*Warileya leponti* Galati and Cáceres 1999.

Distribution: Peru.

*Warileya lumbrerasi* Ogosuku, Perez, Davies and Villaseca, 1996.

Distribution: Peru.

*Warileya nigrosaccula* Fairchild and Hertig, 1951.

Distribution: Colombia, Panama.

*Warileya phlebotomanica* Hertig, 1948.

Distribution: Ecuador, Peru.

*Warileya rotundipennis* Fairchild and Hertig, 1951.

Distribution: Costa Rica, Panama, Colombia, Peru, Bolivia.

*Warileya yungasi* Velasco and Trapido, 1974.

Distribution: Bolivia.

Tribe PHLEBOTOMINI Rondani 1840.

*Edentomyia* Galati, Andrade Filho, Silva and Falcão, 2003 (1 species).

*Edentomyia piauiensis* Galati, Andrade Filho, Silva and Falcão, 2003.

Distribution: Brazil.

Subtribe BRUMPTOMYIINA Galati, 1995.

*Brumptomyia* França and Parrot, 1921 (28 species).

*Brumptomyia angelae* Galati, Santos and Silva, 2007.

Distribution: Brazil.

*Brumptomyia avellari* (Costa Lima, 1932).

Distribution: Argentina, Bolivia, Brazil, Colombia, Panama, Paraguay, Peru, Venezuela.

*Brumptomyia beaupertuyi* (Ortiz, 1954).

Distribution: Brazil, Colombia, Peru, Venezuela.

*Brumptomyia bragai* (Mangabeira and Sherlock, 1961).

Distribution: Brazil.

*Brumptomyia brumpti* (Larrousse, 1920).

Distribution: Argentina, Bolivia, Brazil.

*Brumptomyia cardosoi* (Barretto and Coutinho, 1941).

Distribution: Brazil.

*Brumptomyia carvalheiroi* Shimabukuro, Marassá and Galati, 2007.

Distribution: Brazil.

*Brumptomyia cavicola* Barreto, 1964.

Distribution: Brazil.

*Brumptomyia cunhai* (Mangabeira, 1942).

Distribution: Argentina, Brazil, Honduras.

*Brumptomyia dasipophyla* Barreto, 1964.

Distribution: Brazil.

*Brumptomyia devenanzii* (Ortiz and Scorza, 1963).

Distribution: Venezuela.

*Brumptomyia figueireidoi* Mangabeira and Sherlock, 1961.

Distribution: Brazil.

*Brumptomyia galindoi* (Fairchild and Hertig, 1947).

Distribution: Bolivia, Colombia, Costa Rica, Ecuador, Nicaragua, Panama, Peru.

*Brumptomyia guimaraesi* (Coutinho and Barretto, 1941).

Distribution: Argentina, Brazil, Colombia, Paraguay.

*Brumptomyia hamata* (Fairchild and Hertig, 1947).

Distribution: Belize, Colombia, Ecuador, Mexico, Panama, Peru.

*Brumptomyia leopoldoi* (Rodriguez, 1953).

Distribution: Belize, Colombia, Ecuador, Panama, Peru.

*Brumptomyia mangabeirai* (Barretto and Coutinho, 1941).

Distribution: Brazil.

*Brumptomyia mesai* Sherlock, 1962.

Distribution: Belize, Colombia, Colombia, Honduras, Mexico.

*Brumptomyia nitzulescui* (Costa Lima, 1932).

Distribution: Argentina, Brazil.

*Brumptomyia orlandoi* Fraiha, Shaw and Lainson, 1970.

Distribution: Brazil.

*Brumptomyia ortizi* Martins, Silva and Falcão, 1971.

Distribution: Argentina, Brazil.

*Brumptomyia pentacantha* (Barretto, 1947).

Distribution: Bolivia, Brazil, Colombia, Ecuador, Peru.

*Brumptomyia pintoi* (Costa Lima, 1932).

Distribution: Argentina, Bolivia, Brazil, Colombia, French Guiana, Surinam, Venezuela.

*Brumptomyia quimperi* Galati and Cáceres, 1999.

Distribution: Peru.

*Brumptomyia spinosipes* (Floch and Abonnenc, 1943).

Distribution: Brazil, French Guiana, Panama.

*Brumptomyia travassosi* (Mangabeira, 1942).

Distribution: Brazil, French Guiana, Panama, Surinam.

*Brumptomyia troglodytes* (Lutz, 1922).

Distribution: Brazil, Peru.

*Brumptomyia virgensi* Mangabeira and Sherlock, 1961.

Distribution: Brazil.

*Oligodontomyia* Galati, 1995 (3 species).

*Oligodontomyia isopsi* (Léger and Ferté, 1996).

Distribution: Chile.

*Oligodontomyia oligodonta* (Young, Pérez and Romero, 1985).

Distribution: Peru.

*Oligodontomyia toroensis* (Le Pont, Torrez-Espejo and Dujardin, 1997).

Distribution: Bolivia.

SERGENTOMYIINA Galati, 1995.

*Deanemyia* Galati, 1995 (5 species).

*Deanemyia appendiculata* (Martins, Falcão and Silva, 1961).

Distribution: Brazil.

*Deanemyia derelicta* (Freitas and Barrett, 1999).

Distribution: Brazil.

*Deanemyia maruaga* (Alves, Freitas and Barrett, 2008).

Distribution: Brazil.

*Deanemyia ramirezi* (Martins, Falcão, Silva and Miranda-Filho, 1982).

Distribution: Bolivia, Brazil.

*Deanemyia samueli* (Deane, 1955).

Distribution: Brazil.

*Micropygomyia* Barretto, 1962 (59 species).

(*Coquillettimyia*) Galati, 1995.

Vexator series Fairchild, 1955.

*Micropygomyia* (*Coquillettimyia*) *apache* (Young and Perkins, 1984).

Distribution: United States.

*Micropygomyia* (*Coquilletttimyia*) *nahua* Ibáñez-Bernal, García-Torres, Vásquez-Márquez, 2017.

Distribution: Mexico.

*Micropygomyia* (*Coquillettimyia*) *oppidana* (Dampf, 1944).

Distribution: Canada, Mexico, United States.

*Micropygomyia* (*Coquillettimyia*) *vexator* (Coquillett, 1907).

Distribution: Canada, Mexico, United States.

*Micropygomyia* (*Coquillettimyia*) *vindicator* (Dampf, 1944).

Distribution: Mexico.

Chiapanensis series Theodor, 1965.

*Micropygomyia* (*Coquillettimyia*) *californica* (Fairchild and Hertig, 1957).

Distribution: United States.

*Micropygomyia* (*Coquillettimyia*) *chiapanensis* (Dampf, 1947).

Distribution: Costa Rica, El Salvador, Honduras, Mexico, Nicaragua, Panama.

*Micropygomyia* (*Coquillettimyia*) *stewarti* (Mangabeira and Galindo, 1944).

Distribution: Mexico, United States.

(*Micropygomyia*) *s.s.* Barretto, 1962.

Cayennensis series Fairchild, 1955.

*Micropygomyia* (*Micropygomyia*) *absonodonta* (Feliciangeli, 1995).

Distribution: Peru, Venezuela.

*Micropygomyia* (*Micropygomyia*) *ancashensis* Galati and Cáceres, 2007.

Distribution: Peru.

*Micropygomyia* (*Micropygomyia*) *cayennensis cayennensis* (Floch and Abonnenc, 1941).

Distribution: Belize, Brazil, Colombia, Costa Rica, Ecuador, El Salvador, French Guiana, Honduras, Martinica, Mexico, Nicaragua, Panama, Peru, Trinidad and Tobago, Venezuela.

*Micropygomyia* (*Micropygomyia*) *cayennensis braci* (Lewis, 1967).

Distribution: Cayman Islands.

*Micropygomyia* (*Micropygomyia*) *cayennensis cruzi* (Gonzáles and García, 1981).

Distribution: Cuba.

*Micropygomyia* (*Micropygomyia*) *cayennensis hispaniolae* (Fairchild and Trapido, 1950).

Distribution: Dominican Republic, Haiti.

*Micropygomyia* (*Micropygomyia*) *cayennensis jamaicensis* (Fairchild and Trapido, 1950).

Distribution: Jamaica.

*Micropygomyia* (*Micropygomyia*) *cayennensis maciasi* (Fairchild and Hertig, 1948).

Distribution: Belize, Guatemala, Mexico.

*Micropygomyia* (*Micropygomyia*) *cayennensis puertoricensis* (Fairchild and Hertig, 1948).

Distribution: Puerto Rico.

*Micropygomyia* (*Micropygomyia*) *cayennensis viequesensis* (Fairchild and Hertig, 1948).

Distribution: Puerto Rico, United States Virgin Islands.

*Micropygomyia* (*Micropygomyia*) *ctenidophora* (Fairchild and Hertig, 1948).

Distribution: Mexico.

*Micropygomyia* (*Micropygomyia*) *cubensis* (Fairchild and Trapido, 1950).

Distribution: Cuba, United States.

*Micropygomyia* (*Micropygomyia*) *duppyorum* (Fairchild and Trapido, 1950).

Distribution: Jamaica.

*Micropygomyia* (*Micropygomyia*) *durani* (Vargas and Díaz-Nájera, 1952).

Distribution: El Salvador, Honduras, Mexico.

*Micropygomyia* (*Micropygomyia*) *farilli* (Vargas and Díaz-Nájera, 1959).

Distribution: Mexico.

*Micropygomyia* (*Micropygomyia*) *hardisoni* (Vargas and Díaz-Nájera, 1952).

Distribution: Mexico.

*Micropygomyia* (*Micropygomyia*) *lewisi* (Feliciangeli, Ordoñez and Fernández, 1984).

Distribution: Venezuela.

*Micropygomyia* (*Micropygomyia*) *micropyga* (Mangabeira, 1942).

Distribution: Bolivia, Brazil, Colombia, Costa Rica, Ecuador, French Guyanna, Panama, Peru, Trinidad and Tobago, Venezuela.

*Micropygomyia* (*Micropygomyia*) *schreiberi* (Martins, Falcão and Silva, 1975).

Distribution: Brazil.

*Micropygomyia* (*Micropygomyia*) *wirthi* (Vargas and Díaz-Nájera, 1951).

Distribution: Mexico.

*Micropygomyia* (*Micropygomyia*) *yencanensis* (Ortiz, 1965).

Distribution: Colombia, Venezuela.

Pilosa series Theodor, 1965.

*Micropygomyia* (*Micropygomyia*) *chassigneti* (Floch and Abonnenc, 1944).

Distribution: Brazil, Colombia, French Guiana, Surinam.

*Micropygomyia* (*Micropygomyia*) *mangabeirana* (Martins, Falcão and Silva, 1963).

Distribution: Brazil.

*Micropygomyia* (*Micropygomyia*) *pilosa* (Damasceno and Causey, 1944).

Distribution: Brazil, Colombia, Costa Rica, French Guiana, Panama, Trinidad and Tobago, Venezuela.

(*Sauromyia*) Galati, 1995.

Oswaldoi series Barretto, 1962.

*Micropygomyia* (*Sauromyia*) *capixaba* (Dias, Falcão, Silva and Martins, 1987).

Distribution: Argentina, Brazil.

*Micropygomyia* (*Sauromyia*) *dereuri* (Le Pont, Matias, Martínez and Dujardin 2004).

Distribution: Bolivia, Peru.

†*Micropygomyia* (*Sauromyia*) *dorafeliciangeliae* Andrade Filho, Galati and Brazil, 2009.

Distribution: Dominican Republic.

*Micropygomyia* (*Sauromyia*) *ferreirana* (Barretto, Martins and Pellegrino, 1956).

Distribution: Argentina, Brazil, Peru.

*Micropygomyia* (*Sauromyia*) *huacalquensis* (Le Pont, Matías, Martínez and Dujardin 2004).

Distribution: Bolivia.

*Micropygomyia* (*Sauromyia*) *longipennis* (Barretto, 1946).

Distribution: Brazil, Peru.

*Micropygomyia* (*Sauromyia*) *machupicchu* Martins, Llanos and Silva, 1975.

Distribution: Peru.

*Micropygomyia* (*Sauromyia*) *oswaldoi* (Mangabeira, 1942).

Distribution: Argentina, Bolivia, Brazil.

†*Micropygomyia* (*Sauromyia*) *paterna* (Quate, 1963).

Distribution: Mexico.

*Micropygomyia* (*Sauromyia*) *peresi* (Mangabeira, 1942).

Distribution: Argentina, Bolivia, Brazil, French Guiana.

*Micropygomyia* (*Sauromyia*) *petari* Galati, Marassá and Gonçalves-Andrade, 2003.

Distribution: Brazil.

*Micropygomyia* (*Sauromyia*) *pratti* (Vargas and Díaz-Nájera, 1951).

Distribution: Mexico.

*Micropygomyia* (*Sauromyia*) *pusilla* (Dias, Martins, Falcão and Silva, 1986).

Distribution: Brazil, French Guiana.

*Micropygomyia* (*Sauromyia*) *quechua* (Martins, Llanos and Silva, 1975).

Distribution: Peru.

*Micropygomyia* (*Sauromyia*) *quinquefer* (Dyar, 1929).

Distribution: Argentina, Bolivia, Brazil, Paraguay.

*Micropygomyia* (*Sauromyia*) *rorotaensis* (Floch and Abonnenc, 1944).

Distribution: Brazil, Colombia, French Guiana, Peru, Surinam, Panama, Venezuela.

*Micropygomyia* (*Sauromyia*) *saccai (*Feliciangeli, Ramírez Pérez and Ramírez, 1989).

Distribution: Venezuela.

*Micropygomyia* (*Sauromyia*) *trinidadensis* (Newstead, 1922).

Distribution: Belize, Bolivia, Brazil, Colombia, Costa Rica, Curaçao, El Salvador, French Guiana, Guatemala, Honduras, Mexico, Nicaragua, Peru, Panama, Surinam, Trinidad and Tobago, Venezuela.

*Micropygomyia* (*Sauromyia*) *villelai* (Mangabeira, 1942).

Distribution: Brazil.

*Micropygomyia* (*Sauromyia*) *vonatzingeni* Galati, 2007.

Distribution: Brazil.

*Micropygomyia* (*Sauromyia*) *zikani (*Barretto, 1950).

Distribution: Brazil.

Atroclavata series Fairchild, 1965.

*Micropygomyia* (*Sauromyia*) *atroclavata* (Knab, 1913).

Distribution: Colombia, Costa Rica, Guadalupe, Martinica, Panama, Trinidad and Tobago, Venezuela, United States, Virgin Islands.

*Micropygomyia* (*Sauromyia*) *venezuelensis* (Floch and Abonnenc, 1948).

Distribution: Colombia, Venezuela.

(*Silvamyia*) Galati, 1995.

*Micropygomyia* (*Silvamyia*) *acanthopharynx* (Martins, Falcão and Silva, 1962).

Distribution: Brazil.

*Micropygomyia* (*Silvamyia*) *echinatopharynx* Andrade Filho, Galati, Andrade and Falcão, 2004.

Distribution: Brazil.

*Incertae sedis*

†*Micropygomyia brandaoi* Andrade Filho, Galati, Falcão, Brazil, 2008.

Distribution: Dominican Republic.

*Micropygomyia xerophila* (Young, Brener and Wargo, 1983).

Distribution: United States.

LUTZOMYIINA Abonnenc and Léger, 1976.

*Dampfomyia* Addis, 1945 (20 species).

(*Dampfomyia*) Addis, 1945.

*Dampfomyia* (*Dampfomyia*) *anthophora* (Addis, 1945).

Distribution: Mexico, Nicaragua, United States.

*Dampfomyia* (*Dampfomyia*) *atulapai* (De León, 1971).

Distribution: El Salvador, Guatemala, Mexico.

*Dampfomyia* (*Dampfomyia*) *caminoi* (Young and Duncan, 1994).

Distribution: Mexico.

*Dampfomyia* (*Dampfomyia*) *dodgei* (Vargas and Díaz-Nájera, 1953).

Distribution: El Salvador, Mexico.

*Dampfomyia* (*Dampfomyia*) *insolita* (Fairchild and Hertig, 1956).

Distribution: Costa Rica, Panama.

*Dampfomyia* (*Dampfomyia*) *leohidalgoi* (Ibáñez-Bernal, Hernández-Xoliot and Mendoza, 2006).

Distribution: Mexico.

*Dampfomyia* (*Dampfomyia*) *permira* (Fairchild and Hertig, 1956).

Distribution: Belie, Mexico, Guatemala.

*Dampfomyia* (*Dampfomyia*) *rosabali* (Fairchild and Hertig, 1956).

Distribution: Colombia, Costa Rica, Panama.

(*Coromyia*) Barretto, 1962.

*Dampfomyia* (*Coromyia*) *aquilonia* (Fairchild and Harwood, 1961).

Distribution: Canada, United States.

*Dampfomyia* (*Coromyia*) *beltrani* (Vargas and Díaz-Nájera, 1951).

Distribution: Honduras, Mexico.

*Dampfomyia* (*Coromyia*) *deleoni* (Fairchild and Hertig, 1947).

Distribution: Belize, Costa Rica, El Salvador, Guatemala, Honduras, Mexico.

*Dampfomyia* (*Coromyia*) *disneyi* (Williams, 1987).

Distribution: Belize, Guatemala, Mexico.

*Dampfomyia* (*Coromyia*) *isovespertilionis* (Fairchild and Hertig, 1958).

Distribution: Colombia, Costa Rica, Panama.

*Dampfomyia* (*Coromyia*) *steatopyga* (Fairchild and Hertig, 1958).

Distribution: Mexico.

*Dampfomyia* (*Coromyia*) *vesicifera* (Fairchild and Hertig, 1947).

Distribution: Costa Rica, Nicaragua, Panama.

*Dampfomyia* (*Coromyia*) *vespertilionis* (Fairchild and Hertig, 1947).

Distribution: Colombia, Costa Rica, Ecuador, Nicaragua, Panama.

*Dampfomyia* (*Coromyia*) *viriosa* (Fairchild and Hertig, 1958).

Distribution: Costa Rica, Panama.

*Dampfomyia zeledoni* (Young and Murillo, 1984).

Distribution: Costa Rica, Honduras, Costa Rica.

Delpozoi group.

*Dampfomyia* (*Coromyia*) *delpozoi* (Vargas and Díaz-Nájera, 1953).

Distribution: Belize, Guatemala, Mexico.

*Dampfomyia* (*Coromyia*) *inusitata* (Fairchild and Hertig, 1961).

Distribution: Mexico.

*Evandromyia* Mangabeira, 1941 (47 species).

(*Aldamyia*) Galati, 1995.

*Evandromyia* (*Aldamyia*) *aldafalcaoae* (Santos, Andrade-Filho and Honer, 2001).

Distribution: Argentina, Brazil.

*Evandromyia* (*Aldamyia*) *andersoni* (Le Pont and Desjeux, 1988).

Distribution: Bolivia, Brazil.

*Evandromyia* (*Aldamyia*) *apurinan* Shimabukuro, Figueira and Silva, 2013.

Distribution: Brazil.

*Evandromyia* (*Aldamyia*) *bacula* (Martins, Falcão and Silva, 1965).

Distribution: Bolivia, Brazil.

*Evandromyia* (*Aldamyia*) *carmelinoi* (Ryan, Fraiha, Lainson and Shaw, 1986).

Distribution: Brazil.

*Evandromyia* (*Aldamyia*) *dubitans* (Sherlock, 1962).

Distribution: Brazil, Colombia, Costa Rica, Panama, Trinidad and Tobago, Venezuela.

*Evandromyia* (*Aldamyia*) *evandroi* (Costa Lima and Antunes, 1936).

Distribution: Argentina, Brazil.

*Evandromyia* (*Aldamyia*) *hashiguchii* Leon, Teran, Neira and Le Pont, 2009.

Distribution: Ecuador.

*Evandromyia* (*Aldamyia*) *lenti* (Mangabeira, 1938).

Distribution: Bolivia, Brazil, Surinam.

*Evandromyia* (*Aldamyia*) *orcyi* Oliveira, Sanguinette, Almeida and Andrade Filho, 2015.

Distribution: Brazil.

*Evandromyia* (*Aldamyia*) *piperiformis* Godoy, Cunha and Galati, 2017.

Distribution: Brazil.

*Evandromyia* (*Aldamyia*) *sericea* (Floch and Abonnenc, 1944).

Distribution: Brazil, Colombia, Ecuador, French Guiana, Surinam, Venezuela.

*Evandromyia* (*Aldamyia*) *termitophila* (Martins, Falcão and Silva, 1964).

Distribution: Argentina, Bolivia, Brazil.

*Evandromyia* (*Aldamyia*) *walkeri* (Newstead, 1914).

Distribution: Bolivia/Brazil, Colombia, Ecuador, French Guiana, Panama, Peru, Trinidad and Tobago, Venezuela.

*Evandromyia* (*Aldamyia*) *williamsi* (Damasceno, Causey and Arouck, 1945).

Distribution: Brazil, Peru, Venezuela.

(*Barrettomyia*) Martins and Silva, 1968.

Cortelezzii series Galati, 1955.

*Evandromyia* (*Barrettomyia*) *chacuaensis* Szelag, Rosa, Galati, Andrade Fhilo and Salomón, 2018.

Distribution: Argentina.

*Evandromyia* (*Barrettomyia*) *cortelezzii* (Brèthes, 1923).

Distribution: Argentina, Bolivia, Brazil, Paraguay, Peru, Uruguay.

*Evandromyia* (*Barrettomyia*) *corumbaensis* (Galati, Nunes, Oshiro and Rego, 1989).

Distribution: Argentina, Bolivia, Brazil.

*Evandromyia* (*Barrettomyia*) *cristacapta* Galati, Rosa, Andrade Filho and Salomón, 2021.

Distribution: Argentina.

*Evandromyia* (*Barrettomyia*) *sallesi* (Galvão and Coutinho, 1939).

Distribution: Argentina, Bolivia, Brazil, Ecuador, Paraguay, Peru.

*Evandromyia* (*Barrettomyia*) *spelunca* Carvalho, Brazil, Sanguinette and Andrade-Filho, 2011.

Distribution: Brazil.

Monstruosa series Lewis, Young, Fairchild and Minter, 1977.

*Evandromyia* (*Barrettomyia*) *monstruosa* (Floch and Abonnenc, 1944).

Distribution: Brazil, Colombia, French Guiana, Peru, Surinam, Venezuela.

*Evandromyia* (*Barrettomyia*) *teratodes* (Martins, Falcão and Silva, 1964).

Distribution: Brazil, Paraguay.

Tupynambai series Martins and Silva, 1968.

*Evandromyia* (*Barrettomyia*) *bahiensis* (Mangabeira and Sherlock, 1961).

Distribution: Brazil.

*Evandromyia* (*Barrettomyia*) *callipyga* (Martins and Silva, 1965).

Distribution: Brazil.

*Evandromyia* (*Barrettomyia*) *costalimai* (Mangabeira, 1942).

Distribution: Brazil.

*Evandromyia* (*Barrettomyia*) *petropolitana* (Martins and Silva, 1968).

Distribution: Brazil.

*Evandromyia* (*Barrettomyia*) *tupynambai* (Mangabeira, 1942).

Distribution: Brazil.

(*Evandromyia*) *s.s*. Mangabeira, 1941.

Infraspinosa series Young and Arias, 1977.

*Evandromyia* (*Evandromyia*) *begonae* (Ortiz and Torrez, 1975).

Distribution: Brazil, Colombia, Venezuela.

*Evandromyia* (*Evandromyia*) *bourrouli* (Barretto and Coutinho,1941).

Distribution: Argentina, Bolivia, Brazil.

*Evandromyia* (*Evandromyia*) *brachyphalla* (Mangabeira, 1941).

Distribution: Brazil, French Guiana.

*Evandromyia* (*Evandromyia*) *georgii* (Freitas and Barrett, 2002).

Distribution: Brazil.

*Evandromyia* (*Evandromyia*) *infraspinosa* (Mangabeira, 1941).

Distribution: Bolivia, Brazil, Colombia, French Guiana, Peru, Surinam, Venezuela.

*Evandromyia* (*Evandromyia*) *inpai* (Young and Arias, 1977).

Distribution: Brazil, Venezuela.

*Evandromyia* (*Evandromyia*) *ledezmaae* Leon, Teran, Neira and Le Pont, 2009.

Distribution: Ecuador.

*Evandromyia* (*Evandromyia*) *pinottii* (Damasceno and Arouck, 1956).

Distribution: Brazil, French Guiana, Peru, Venezuela.

*Evandromyia* (*Evandromyia*) *sipani* (Fernández, Carbajal, Alexander and Need, 1994).

Distribution: Brazil, Colombia, Peru.

*Evandromyia* (*Evandromyia*) *tarapacaensis* (Le Pont, Torrez-Espejo and Galati, 1997).

Distribution: Bolivia, Brazil.

Rupicola series Young and Fairchild, 1974.

*Evandromyia* (*Evandromyia*) *correalimai* (Martins, Coutinho and Luz, 1970).

Distribution: Brazil.

*Evandromyia* (*Evandromyia*) *gaucha* Andrade-Filho, Souza and Falcão, 2007.

Distribution: Brazil.

*Evandromyia* (*Evandromyia*) *grimaldii* Andrade-Filho, Pinto, Santos and Carvalho, 2009.

Distribution: Brazil.

*Evandromyia* (*Evandromyia*) *rupicola* (Martins, Godoy and Silva, 1962).

Distribution: Brazil.

*Evandromyia* (*Evandromyia*) *tylophalla* Andrade and Galati, 2012.

Distribution: Brazil.

Saulensis series Lewis et al. 1977.

*Evandromyia* (*Evandromyia*) *saulensis* (Floch and Abonnenc, 1944).

Distribution: Bolivia, Brazil, Colombia, Costa Rica, Ecuador, French Guiana, Panama, Peru, Venezuela.

*Evandromyia* (*Evandromyia*) *wilsoni* (Damasceno and Causey, 1945).

Distribution: Brazil.

*Incertae sedis*

*Evandromyia diamantinensis* (Barata, Serra e Meira and Carvalho, 2012).

Distribution: Brazil.

*Evandromyia edwardsi* (Mangabeira, 1941).

Distribution: Argentina, Brazil.

*Expapillata* Galati, 1995 (2 species).

*Expapillata cerradincola* (Galati, Nunes, Oshiro and Dorval, 1995).

Distribution: Bolivia, Brazil.

*Expapillata firmatoi* (Barretto, Martins and Pellegrino, 1956).

Distribution: Argentina, Brazil.

*Lutzomyia* França, 1924 (82 species).

(*Castromyia*) Mangabeira, 1942.

*Lutzomyia* (*Castromyia*) *amarali* (Barretto and Coutinho, 1940).

Distribution: Brazil.

*Lutzomyia* (*Castromyia*) *caligata* Martins, Falcão and Silva, 1965.

Distribution: Brazil.

*Lutzomyia* (*Castromyia*) *castroi* (Barretto and Coutinho, 1941).

Distribution: Brazil.

(*Helcocyrtomyia*) Barretto, 1962.

Osornoi series Galati and Cáceres, 1994.

*Lutzomyia* (*Helcocyrtomyia*) *caballeroi* Blancas, Cáceres and Galati, 1989.

Distribution: Peru.

*Lutzomyia* (*Helcocyrtomyia*) *castanea* Galati and Cáceres, 1994.

Distribution: Ecuador, Peru.

*Lutzomyia* (*Helcocyrtomyia*) *ceferinoi* (Ortiz and Alvarez, 1963).

Distribution: Colombia, Venezuela.

*Lutzomyia* (*Helcocyrtomyia*) *erwindonaldoi* (Ortiz, 1978).

Distribution: Colombia, Venezuela.

*Lutzomyia* (*Helcocyrtomyia*) *herreri* Galati and Cáceres, 2003.

Distribution: Peru.

*Lutzomyia* (*Helcocyrtomyia*) *imperatrix* (Alexander, 1944).

Distribution: Peru.

*Lutzomyia* (*Helcocyrtomyia*) *larensis* Arredondo, 1987.

Distribution: Venezuela.

*Lutzomyia* (*Helcocyrtomyia*) *munaypata* Ogusuku, Chevarria, Porras and Pérez, 1999Distribution: Brazil, Peru.

*Lutzomyia* (*Helcocyrtomyia*) *osornoi* (Ristorcelli and Van Ty, 1941).

Distribution: Bolivia, Colombia, Ecuador, Peru.

*Lutzomyia* (*Helcocyrtomyia*) *quillabamba* Ogusuku, Chevarria, Porras and Pérez, 1999.

Distribution: Peru.

*Lutzomyia* (*Helcocyrtomyia*) *rispaili* Torrez-Espejo, Cáceres and Le Pont, 1995.

Distribution: Bolivia, Peru.

*Lutzomyia* (*Helcocyrtomyia*) *strictivilla* Young, 1979.

Distribution: Colombia, Ecuador, Venezuela.

*Lutzomyia* (*Helcocyrtomyia*) *wattsi* Fernández, Carbajal, Astete and Wooster, 1998.

Distribution: Peru.

Peruensis series Barretto, 1962.

*Lutzomyia* (*Helcocyrtomyia*) *ayacuchensis* Cáceres and Galati, 1988.

Distribution: Ecuador, Peru.

*Lutzomyia* (*Helcocyrtomyia*) *blancasi* Galati and Cáceres, 1990.

Distribution: Peru.

*Lutzomyia* (*Helcocyrtomyia*) *chavinensis* Pérez and Ogusuku, 1999.

Distribution: Peru.

*Lutzomyia* (*Helcocyrtomyia*) *galatiae* Le Pont, Martinez, Torrez-Espejo and Dujardin, 1998.

Distribution: Bolivia.

*Lutzomyia* (*Helcocyrtomyia*) *noguchii* (Shannon, 1929).

Distribution: Peru.

*Lutzomyia* (*Helcocyrtomyia*) *pallidithorax* Galati and Cáceres, 1994.

Distribution: Peru.

*Lutzomyia* (*Helcocyrtomyia*) *peruensis* (Shannon, 1929).

Distribution: Bolivia, Peru.

*Lutzomyia* (*Helcocyrtomyia*) *pescei* (Hertig, 1943).

Distribution: Peru.

*Lutzomyia* (*Helcocyrtomyia*) *tejadai* Galati and Cáceres, 1990.

Distribution: Peru.

Sanguinaria series Barretto, 1962.

*Lutzomyia* (*Helcocyrtomyia*) *adamsi* Fernandez, Galati, Carbajal, Wooster and Watts, 1998.

Distribution: Peru.

*Lutzomyia* (*Helcocyrtomyia*) *botella* (Fairchild and Hertig, 1961).

Distribution: Panama.

*Lutzomyia* (*Helcocyrtomyia*) *caceresi* Le Pont, Matías, Martínez and Dujardin, 2004.

Distribution: Bolivia.

*Lutzomyia* (*Helcocyrtomyia*) *cirrita* (Young and Porter, 1974).

Distribution: Colombia.

*Lutzomyia* (*Helcocyrtomyia*) *gonzaloi* Ogusuku Canales and Pérez, 1997.

Distribution: Brazil, Peru.

*Lutzomyia* (*Helcocyrtomyia*) *guderiani* Torrez-Espejo, Cáceres and Le Pont, 1995.

Distribution: Bolivia, Peru.

*Lutzomyia* (*Helcocyrtomyia*) *hartmanni* (Fairchild and Hertig, 1957).

Distribution: Colombia, Costa Rica, Ecuador, Mexico, Panama, Peru.

*Lutzomyia* (*Helcocyrtomyia*) *kirigetiensis* Galati and Cáceres, 1992.

Distribution: Brazil, Peru.

*Lutzomyia* (*Helcocyrtomyia*) *monzonensis* Ogusuku, Canales and Pérez, 1997.

Distribution: Peru.

*Lutzomyia* (*Helcocyrtomyia*) *sanguinaria* (Fairchild and Hertig, 1957).

Distribution: Colombia, Costa Rica, Ecuador, Panama.

*Lutzomyia* (*Helcocyrtomyia*) *scorzai* (Ortiz, 1965).

Distribution: Colombia, Peru, Venezuela.

*Lutzomyia* (*Helcocyrtomyia*) *tolimensis* Carrasquilla, Munstermann, Marín, Ocampo and Ferro, 2012.

Distribution: Colombia.

*Lutzomyia* (*Helcocyrtomyia*) *tortura* Young and Rogers, 1984.

Distribution: Bolivia, Colombia, Ecuador.

*Lutzomyia* (*Helcocyrtomyia*) *velezi* Bejarano, Vivero and Uribe, 2010.

Distribution: Colombia.

*Incertae sedis*

*Lutzomyia infusca* Porter and Young, 1999.

Distribution: Guatemala.

*Lutzomyia* (*Helcocyrtomyia*) *vargasi* (Fairchild and Hertig, 1961) .

Distribution: Mexico.

(*Lutzomyia*) França, 1924.

*Lutzomyia* (*Lutzomyia*) *alencari* Martins, Souza and Falcão, 1962.

Distribution: Brazil.

*Lutzomyia* (*Lutzomyia*) *almerioi* Galati and Nunes, 1999.

Distribution: Brazil, Paraguay.

*Lutzomyia* (*Lutzomyia*) *battistinii* (Hertig, 1943).

Distribution: Peru.

*Lutzomyia* (*Lutzomyia*) *bicornuta* (Blancas and Herrer, 1959/1960).

Distribution: Peru.

*Lutzomyia* (*Lutzomyia*) *bifoliata* Osorno-Mesa, Morales, Osorno and Hoyos, 1970.

Distribution: Colombia.

*Lutzomyia* (*Lutzomyia*) *cavernicola* (Costa Lima, 1932).

Distribution: Brazil.

*Lutzomyia* (*Lutzomyia*) *cruzi* (Mangabeira, 1938).

Distribution: Bolivia, Brazil.

*Lutzomyia* (*Lutzomyia*) *dispar* Martins and Silva, 1963.

Distribution: Brazil.

*Lutzomyia* (*Lutzomyia*) *elizabethrangelae* Vilela, Azevedo and Godoy, 2015.

Distribution: Brazil.

*Lutzomyia* (*Lutzomyia*) *falquetoi* Pinto and Santos, 2007.

Distribution: Brazil.

*Lutzomyia fonsecai* (Costa Lima, 1932).

Distribution: Bolivia.

*Lutzomyia* (*Lutzomyia*) *forattinii* Galati, Rego, Nunes and Teruya, 1985.

Distribution: Bolivia, Brazil.

*Lutzomyia* (*Lutzomyia*) *gaminarai* (Cordero, Vogelsang and Cossio, 1928).

Distribution: Brazil, Paraguay, Uruguay.

*Lutzomyia* (*Lutzomyia*) *ischnacantha* Martins, Souza and Falcão, 1962.

Distribution: Brazil.

*Lutzomyia* (*Lutzomyia*) *ischyracantha* Martins, Falcão and Silva, 1962.

Distribution: Brazil.

*Lutzomyia* (*Lutzomyia*) *itambe* Chaves Júnior, Lima, Paranhos and Andrade, 2023.

Distribution: Brazil.

*Lutzomyia* (*Lutzomyia*) *lichyi* (Floch and Abonnenc, 1950).

Distribution: Brazil, Colombia, Costa Rica, Ecuador, French Guiana, Panama, Peru, Trinidad and Tobago, Venezuela.

*Lutzomyia* (*Lutzomyia*) *longipalpis* (Lutz and Neiva, 1912).

Distribution: Argentina, Bolivia, Brazil, Colombia, Costa Rica, El Salvador, Guatemala, Honduras, Mexico, Nicaragua, Panama, Paraguay, Uruguay, Venezuela.

*Lutzomyia* (*Lutzomyia*) *matiasi* Le Pont and Mollinedo, 2009.

Distribution: Bolivia.

*Lutzomyia* (*Lutzomyia*) *pseudolongipalpis* Arrivillaga and Feliciangeli 2001.

Distribution: Venezuela.

*Lutzomyia* (*Lutzomyia*) *renei* (Martins, Falcão and Silva, 1957).

Distribution: Brazil.

*Lutzomyia* (*Lutzomyia*) *souzalopesi* Martins, Silva and Falcão, 1970.

Distribution: Brazil.

(*Tricholateralis*) Galati, 1995.

*Lutzomyia* (*Tricholateralis*) *araracuarensis* Morales and Minter, 1981.

Distribution: Brazil, Colombia, Peru.

*Lutzomyia* (*Tricholateralis*) *carvalhoi* Damasceno, Causey and Arouck, 1945.

Distribution: Brazil, French Guiana.

*Lutzomyia* (*Tricholateralis*) *cruciata* Coquillett, 1907.

Distribution: Belize, Brazil, Costa Rica, El Salvador, Guatemala, Honduras, Mexico, Nicaragua, Panama, United States.

*Lutzomyia* (*Tricholateralis*) *cultellata* Freitas and Albuquerque, 1996.

Distribution: Brazil, Peru.

*Lutzomyia* (*Tricholateralis*) *diabolica* Hall, 1936.

Distribution: Mexico, United States.

*Lutzomyia* (*Tricholateralis*) *evangelistai* Martins and Fraiha, 1971.

Distribution: Bolivia, Brazil, Colombia, Peru.

*Lutzomyia* (*Tricholateralis*) *falcata* Young, Morales and Ferro, 1994.

Distribution: Brazil, Colombia, Ecuador, Peru.

*Lutzomyia* (*Tricholateralis*) *flabellata* Martins and Silva, 1964.

Distribution: Bolivia, Brazil, Peru.

*Lutzomyia* (*Tricholateralis*) *gomezi* (Nitzulescu, 1931).

Distribution: Bolivia, Brazil, Colombia, Costa Rica, Ecuador, El Salvador, French Guyana, Honduras, Mexico, Nicaragua, Panama, Peru, Trinidad and Tobago, Venezuela.

*Lutzomyia* (*Tricholateralis*) *legerae* Le Pont, Gantier, Hue and Valle, 1995.

Distribution: Nicaragua.

*Lutzomyia* (*Tricholateralis*) *maesi* Le Pont, Ibáñez-Bernal and Fuentes, 2011.

Distribution: Nicaragua.

*Lutzomyia* (*Tricholateralis*) *marinkellei* Young, 1979.

Distribution: Brazil, Colombia.

*Lutzomyia* (*Tricholateralis*) *sherlocki* Martins, Silva and Falcão 1971.

Distribution: Bolivia, Brazil, Colombia, Ecuador, Peru.

*Lutzomyia* (*Tricholateralis*) *spathotrichia* Martins, Falcão and Silva, 1963.

Distribution: Bolivia, Brazil, Ecuador, French Guiana, Peru.

*Incertae sedis*

*Lutzomyia chotensis* Galati, Cáceres and Zorilla, 2003.

Distribution: Peru.

*Lutzomyia ignacioi* (Young, 1972).

Distribution: Colombia, Venezuela.

*Lutzomyia manciola* Ibáñez-Bernal, 2001.

Distribution: Belize, Mexico.

*Lutzomyia ponsi* (Perruollo, 1984).

Distribution: Venezuela.

*Lutzomyia tanyopsis* Young and Perkins, 1984.

Distribution: United States.

*Migonemyia* Galati, 1995 (7 species).

(*Blancasmyia*) Galati, 1995.

*Migonemyia* (*Blancasmyia*) *bursiformis* (Floch and Abonnenc, 1944).

Distribution: Brazil, Colombia, French Guiana, Peru, Venezuela.

*Migonemyia* (*Blancasmyia*) *cerqueirai* (Causey and Damasceno, 1945).

Distribution: Brazil, Colombia, Peru.

*Migonemyia* (*Blancasmyia*) *gorbitzi* (Blancas, 1959/1960).

Distribution: Colombia, Costa Rica, Ecuador, Panama, Peru.

*Migonemyia* (*Blancasmyia*) *moucheti* (Pajot and Le Pont, 1978).

Distribution: Brazil, French Guiana, Peru.

(*Migonemyia)* s.str.

*Migonemyia* (*Migonemyia*) *migonei* (França, 1920).

Distribution: Argentina, Bolivia, Brazil, Colombia, Paraguay, Peru, Trinidad and Tobago, Venezuela.

*Migonemyia* (*Migonemyia*) *rabelloi* (Galati and Gomes, 1992).

Distribution: Brazil.

*Migonemyia* (*Migonemyia*) *vaniae* Galati, Fonseca and Marassá, 2007.

Distribution: Brazil.

*Pintomyia* Costa Lima, 1932 (79 species).

(*Pintomyia*) *s.s*. Costa Lima, 1932.

*Pintomyia* (*Pintomyia*) *bianchigalatiae* (Andrade-Filho, Aguiar, Dias and Falcão, 1999).

Distribution: Argentina, Brazil.

*Pintomyia* (*Pintomyia*) *christenseni* (Young and Duncan, 1994).

Distribution: Brazil, Colombia, Panama, Peru, Trinidad and Tobago, Venezuela.

*Pintomyia* (*Pintomyia*) *damascenoi* (Mangabeira, 1941).

Distribution: Brazil, Colombia, French Guiana, Surinam.

*Pintomyia* (*Pintomyia*) *fischeri* (Pinto, 1926).

Distribution: Argentina, Bolivia, Brazil, Colombia, Paraguay, Peru, Venezuela.

*Pintomyia* (*Pintomyia*) *gibsoni* (Pifano and Ortiz, 1972).

Distribution: Venezuela.

*Pintomyia* (*Pintomyia*) *kuscheli* (Le Pont, Martinez, Torrez-Espejo and Dujardin, 1998).

Distribution: Bolivia, Brazil.

*Pintomyia* (*Pintomyia*) *mamedei* (Oliveira, Afonso, Dias and Brazil, 1994).

Distribution: Brazil.

*Pintomyia* (*Pintomyia*) *pessoai* (Coutinho and Barretto, 1940).

Distribution: Argentina, Brazil, Paraguay.

(*Pifanomyia*) Ortiz and Scorza, 1963.

Evansi series Galati, 1955.

*Pintomyia* (*Pifanomyia*) *evansi* (Nuñez-Tovar, 1924).

Distribution: Colombia, Costa Rica, El Salvador, Guatemala, Honduras, Mexico, Nicaragua, Peru, Venezuela.

*Pintomyia* (*Pifanomyia*) *maranonensis* (Galati, Cáceres and Le Pont, 1995).

Distribution: Ecuador, Peru.

*Pintomyia* (*Pifanomyia*) *nevesi* (Damasceno and Arouck, 1956).

Distribution: Bolivia, Brazil, Colombia, Ecuador, Peru.

*Pintomyia* (*Pifanomyia*) *ovallesi* (Ortiz, 1952).

Distribution: Belize, Colombia, Costa Rica, Guatemala, Honduras, Mexico, Nicaragua, Panama, Trinidad and Tobago, Venezuela.

*Pintomyia* (*Pifanomyia*) *veintemillasi* Martinez, Leon, Mihalca, Dujardin and Le Pont, 2022.

Distribution: Bolivia.

Monticola series Artemiev, 1991.

*Pintomyia* (*Pifanomyia*) *misionensis* (Castro, 1959).

Distribution: Argentina, Brazil, Paraguay.

*Pintomyia* (*Pifanomyia*) *monticola* (Costa Lima, 1932).

Distribution: Argentina, Brazil, Paraguay, Peru.

Pacae series Galati, 1955.

*Pintomyia* (*Pifanomyia*) *gruta* (Ryan, 1986).

Distribution: Brazil.

*Pintomyia* (*Pifanomyia*) *pacae* (Floch and Abonnenc, 1943).

Distribution: Brazil, French Guiana, Surinam.

Pia series Galati, 1955.

*Pintomyia* (*Pifanomyia*) *emberai* (Bejarano, Duque and Vélez, 2004).

Distribution: Colombia.

*Pintomyia* (*Pifanomyia*) *limafalcaoae* Wolff and Galati, 2002.

Distribution: Colombia.

*Pintomyia* (*Pifanomyia*) *pastorae* (Traviezo-Valles, 2019).

Distribution: Venezuela.

*Pintomyia* (*Pifanomyia*) *pia* (Fairchild and Hertig, 1961).

Distribution: Bolivia, Colombia, Costa Rica, Panama, Peru, Venezuela.

*Pintomyia* (*Pifanomyia*) *reclusa* (Fernández and Rogers, 1991).

Distribution: Peru.

*Pintomyia* (*Pifanomyia*) *suapiensis* (Le Pont, Torrez-Espejo and Dujardin, 1997).

Distribution: Bolivia, Peru.

*Pintomyia* (*Pifanomyia*) *tihuiliensis* (Le Pont, Torrez-Espejo and Dujardin, 1997).

Distribution: Bolivia, Colombia, Peru.

*Pintomyia* (*Pifanomyia*) *tocaniensis* (Le Pont, Torrez-Espejo and Dujardin, 1997).

Distribution: Bolivia, Peru.

*Pintomyia* (*Pifanomyia*) *torrealbai* (Martins, Fernandez and Falcão, 1979).

Distribution: Venezuela.

*Pintomyia* (*Pifanomyia*) *valderramai* (Cazorla, 1988).

Distribution: Venezuela.

Serrana series Barretto, 1962.

*Pintomyia* (*Pifanomyia*) *boliviana* (Velasco and Trapido, 1974).

Distribution: Bolivia.

*Pintomyia* (*Pifanomyia*) *christophei* (Fairchild and Trapido, 1950).

Distribution: Dominican Republic, Haiti.

*Pintomyia* (*Pifanomyia*) *diazi* (Gonzales and García, 1981).

Distribution: Cuba.

*Pintomyia* (*Pifanomyia*) *duckei* Oliveira, Alencar and Freitas, 2018.

Distribution: Brazil.

*Pintomyia* (*Pifanomyia*) *fiocruzi* Pereira-Júnior, Pessoa, Marialva and Medeiros, 2019.

Distribution: Brazil.

*Pintomyia* (*Pifanomyia*) *guilvardae* (Le Pont, Martinez, Torrez-Espejo and Dujardin, 1998).

Distribution: Bolivia, Peru.

*Pintomyia* (*Pifanomyia*) *novoae* (Gonzales and García, 1981).

Distribution: Cuba.

*Pintomyia* (*Pifanomyia*) *odax* (Fairchild and Hertig, 1961).

Distribution: Brazil, Costa Rica, French Guiana, Guatemala, Honduras, Nicaragua, Panama, Venezuela.

*Pintomyia* (*Pifanomyia*) *oresbia* (Fairchild and Hertig, 1961).

Distribution: Colombia, Costa Rica, Panama.

*Pintomyia* (*Pifanomyia*) *orestes* (Fairchild and Trapido, 1950).

Distribution: Brazil, Cuba, Cayman Islands.

*Pintomyia* (*Pifanomyia*) *ottolinai* (Ortiz and Scorza, 1963).

Distribution: Venezuela.

*Pintomyia* (*Pifanomyia*) *piedraferroi* (De León, 1971).

Distribution: Guatemala.

*Pintomyia* (*Pifanomyia*) *robusta* (Galati, Cáceres and Le Pont, 1995).

Distribution: Ecuador, Peru.

*Pintomyia* (*Pifanomyia*) *salomoni* Fuenzalida and Quintana, 2019.

Distribution: Argentina.

*Pintomyia* (*Pifanomyia*) *serrana* (Damasceno and Arouck, 1949).

Distribution: Belize, Bolivia, Brazil, Colombia, Costa Rica, Ecuador, French Guiana, Guatemala, Honduras, Mexico, Nicaragua, Panama, Peru, Venezuela.

*Pintomyia* (*Pifanomyia*) *torresi* (Le Pont and Desjeux, 1991).

Distribution: Argentina, Bolivia.

Townsendi series Galati, 1955.

*Pintomyia* (*Pifanomyia*) *amilcari* (Arredondo, 1984).

Distribution: Venezuela.

*Pintomyia* (*Pifanomyia*) *longiflocosa* (Osorno-Mesa, Morales, Osorno and Hoyos, 1970).

Distribution: Colombia.

*Pintomyia* (*Pifanomyia*) *nadiae* (Feliciangeli, Arredondo and Ward, 1992).

Distribution: Venezuela.

†*Pintomyia* (*Pifanomyia*) *paleotownsendi* Andrade Filho, Falcão, Galati and Brazil, 2006.

Distribution: Dominican Republic.

†*Pintomyia* (*Pifanomyia*) *paleotrichia* Andrade Filho, Brazil, Falcão and Galati, 2007.

Distribution: Dominican Republic.

*Pintomyia* (*Pifanomyia*) *quasitownsendi* (Osorno, Osorno-Mesa and Morales, 1972).

Distribution: Colombia.

*Pintomyia* (*Pifanomyia*) *sauroida* (Osorno-Mesa, Morales and Osorno, 1972).

Distribution: Colombia, Venezuela.

*Pintomyia* (*Pifanomyia*) *spinicrassa* (Morales, Osorno-Mesa, Osorno and Hoyos, 1969).

Distribution: Colombia, Venezuela.

*Pintomyia* (*Pifanomyia*) *torvida* (Young, Morales and Ferro, 1994).

Distribution: Colombia.

*Pintomyia* (*Pifanomyia*) *townsendi* (Ortiz, 1959).

Distribution: Colombia, Venezuela.

*Pintomyia* (*Pifanomyia*) *youngi* (Feliciangeli and Murillo, 1985).

Distribution: Colombia, Costa Rica, Venezuela.

Verrucarum series Fairchild, 1955.

*Pintomyia* (*Pifanomyia*) *andina* (Osorno, Osorno-Mesa and Morales, 1972).

Distribution: Colombia.

*Pintomyia* (*Pifanomyia*) *antioquiensis* Wolff and Galati, 2002.

Distribution: Colombia.

*Pintomyia* (*Pifanomyia*) *aulari* (Feliciangeli, Ordoñez and Manzanilla, 1984).

Distribution: Venezuela.

*Pintomyia* (*Pifanomyia*) *cajamarcensis* (Galati, Cáceres and Le Pont, 1995).

Distribution: Peru.

*Pintomyia* (*Pifanomyia*) *columbiana* (Ristorcelli and Van Ty, 1941).

Distribution: Colombia.

*Pintomyia* (*Pifanomyia*) *deorsa* (Pérez, Ogusuku, Monje and Young, 1991).

Distribution: Peru.

*Pintomyia* (*Pifanomyia*) *disjuncta* (Morales, Osorno and Osorno-Mesa, 1974).

Distribution: Colombia.

*Pintomyia* (*Pifanomyia*) *itza* Ibáñez-Bernal, May-Uc and Rebollar-Téllez, 2010.

Distribution: Mexico.

*Pintomyia* (*Pifanomyia*) *moralesi* (Young, 1979).

Distribution: Colombia.

*Pintomyia* (*Pifanomyia*) *verrucarum* (Townsend, 1913).

Distribution: Peru.

*Incertae sedis*

†*Pintomyia adiketis* Poinar, 2008.

Distribution: Dominican Republic.

†*Pintomyia bolontikui* Ibáñez-Bernal, Kraemer, Stebner and Wagner, 2013.

Distribution: Mexico.

†*Pintomyia brazilorum* Andrade Filho, Galati and Falcão 2006.

Distribution: Dominican Republic.

†*Pintomyia dissimilis* Andrade Filho, Serra and Meira, Sanguinette and Brazil, 2009.

Distribution: Dominican Republic.

†*Pintomyia dominicana* Andrade Filho, Galati and Brazil, 2009.

Distribution: Dominican Republic.

†*Pintomyia falcaorum* Brazil and Andrade Filho, 2002.

Distribution: Dominican Republic.

†*Pintomyia filipalpis* (Peñalver and Grimaldi, 2005).

Distribution: Dominican Republic.

†*Pintomyia killickorum* Andrade Filho and Brazil 2004.

Distribution: Dominican Republic.

*Pintomyia maracayensis* (Nuñez-Tovar, 1924).

Distribution: Venezuela.

†*Pintomyia miocena* (Peñalver and Grimaldi, 2005).

Distribution: Dominican Republic.

*Pintomyia naiffi* (Freitas and Oliveira, 2013).

Distribution: Brazil, Peru.

*Pintomyia nuneztovari* (Ortiz, 1954).

Distribution: Bolivia, Colombia, Guatemala, Honduras, Panama, Peru, Venezuela.

†*Pintomyia paleopestis* (Peñalver and Grimaldi, 2005).

Distribution: Dominican Republic.

*Pintomyia rangeliana* (Ortiz, 1953).

Distribution: Colombia, Panama, Trinidad and Togado, Venezuela.

†*Pintomyia succini* (Peñalver and Grimaldi, 2005).

Distribution: Dominican Republic.

*Pressatia* Mangabeira, 1942 (8 species).

*Pressatia calcarata* (Martins and Silva, 1964).

Distribution: Bolivia, Brazil, Peru, Venezuela.

*Pressatia camposi* (Rodríguez, 1950).

Distribution: Colombia, Costa Rica, Ecuador, Nicaragua, Panama.

*Pressatia choti* (Floch and Abonnenc, 1941).

Distribution: Bolivia, Brazil, Colombia, Ecuador, French Guiana, Peru, Surinam.

*Pressatia duncanae* (Le Pont, Martinez, Torrez-Espejo and Durjardin, 1998).

Distribution: Bolivia, Brazil, Colombia, Peru.

*Pressatia dysponeta* (Fairchild and Hertig, 1952).

Distribution: Bolivia, Brazil, Colombia, Costa Rica, Ecuador, Panama, Venezuela.

*Pressatia equatorialis* (Mangabeira, 1942).

Distribution: Brazil, French Guiana.

*Pressatia triacantha* (Mangabeira, 1942).

Distribution: Brazil, Colombia, Ecuador, French Guiana, Peru, Venezuela.

*Pressatia trispinosa* (Mangabeira, 1942).

Distribution: Brazil, French Guiana, Peru.

*Sciopemyia* Barretto, 1962 (12 species).

*Sciopemyia apicalis* Chaves Júnior, Shimabukuro and Andrade, 2022.

Distribution: Brazil.

*Sciopemyia birali* Chaves Júnior, Pinto, Shimabukuro and Andrade, 2022.

Distribution: Brazil.

*Sciopemyia dantastorresi* Chaves Júnior, Shimabukuro and Andrade, 2022.

Distribution: Brazil.

*Sciopemyia fluviatilis* (Floch and Abonnenc, 1944).

Distribution: Brazil, French Guiana.

*Sciopemyia microps* (Mangabeira, 1942).

Distribution: Brazil.

*Sciopemyia nematoducta* (Young and Arias, 1984).

Distribution: Brazil, Colombia.

*Sciopemyia pennyi* (Arias and Freitas, 1981).

Distribution: Brazil.

*Sciopemyia preclara* (Young and Arias, 1984).

Distribution: Bolivia, Brazil, Colombia, Peru.

*Sciopemyia servulolimai* (Damasceno and Causey, 1945).

Distribution: Bolivia, Brazil, Peru.

*Sciopemyia shimabikuroae* Chaves Júnior and Andrade, 2022.

Distribution: Brazil.

*Sciopemyia sordellii* (Shannon and Del Ponte, 1927).

Distribution: Argentina, Bolivia, Brazil, Colombia, Costa Rica, Ecuador, French Guiana, Panama, Peru, Trinidad and Tobago, Venezuela.

*Sciopemyia vattierae* (Le Pont and Desjeux, 1992).

Distribution: Bolivia, Brazil, Colombia, Peru.

*Trichopygomyia* Barretto, 1962 (16 species).

*Trichopygomyia conviti* (Ramirez-Pérez, Martins and Ramirez, 1976).

Distribution: Brazil, Colombia, Venezuela.

*Trichopygomyia dasypodogeton* (Castro, 1939).

Distribution: Bolivia, Brazil.

*Trichopygomyia depaquiti* (Gantier, Gaborit and Rabarison, 2006).

Distribution: Brazil, French Guiana.

*Trichopygomyia elegans* (Martins, Llanos and Silva, 1976).

Distribution: Brazil, Peru.

*Trichopygomyia ferroae* (Young and Morales, 1987).

Distribution: Colombia.

*Trichopygomyia gantieri* (Le Pont and Desjeux, 1987).

Distribution: Bolivia, Peru.

*Trichopygomyia longispina* (Mangabeira, 1942).

Distribution: Brazil, Colombia, French Guiana, Venezuela.

*Trichopygomyia martinezi* (Young and Morales, 1987).

Distribution: Colombia.

*Trichopygomyia pinna* (Feliciangeli, Ramirez-Pérez and Ramirez, 1989).

Distribution: Brazil, Venezuela.

*Trichopygomyia ratcliffei* (Arias, Ready and Freitas, 1983).

Distribution: Brazil.

*Trichopygomyia rondoniensis* (Martins, Falcão and Silva, 1965).

Distribution: Bolivia, Brazil.

*Trichopygomyia trichopyga* (Floch and Abonnenc, 1945).

Distribution: Brazil, French Guiana, Surinam.

*Trichopygomyia triramula* (Fairchild and Hertig, 1952).

Distribution: Belize, Colombia, Costa Rica, Ecuador, Guatemala, Mexico, Panama.

*Trichopygomyia turelli* (Fernández, Galati, Carbajal and Watts, 1998).

Distribution: Peru.

*Trichopygomyia wagleyi* (Causey and Damasceno, 1945).

Distribution: Bolivia, Brazil, Colombia, Venezuela.

*Trichopygomyia witoto* (Young and Morales, 1987).

Distribution: Colombia, Ecuador.

PSYCHODOPYGINA Galati, 1995.

*Bichromomyia* Galati, 1995 (5 species).

*Bichromomyia bicolor* (Fairchild and Theodor, 1971).

Distribution: Brazil, Colombia, Costa Rica, Ecuador, Panama, Peru, Venezuela *Bichromomyia flaviscutellata* (Mangabeira, 1942).

Distribution: Bolivia, Brazil, Colombia, Ecuador, French Guiana, Peru, Surinam, Trinidad and Tobago, Venezuela.

*Bichromomyia nociva* (Young and Arias, 1982).

Distribution: Brazil, Peru.

*Bichromomyia olmeca* (Vargas and Díaz-Nájera, 1959).

Distribution: Belize, Costa Rica, Guatemala, Honduras, Mexico, Nicaragua.

*Bichromomyia reducta* (Feliciangeli, Ramirez Pérez and Ramirez, 1988).

Distribution: Brazil, Colombia, Peru, Venezuela.

*Martinsmyia* Galati, 1995 (11 species).

Alphabetica group Fairchild, 1955.

*Martinsmyia alphabetica* (Fonseca, 1936).

Distribution: Argentina, Brazil, Paraguay.

*Martinsmyia brisolai* (Le Pont and Desjeux, 1987).

Distribution: Bolivia, Brazil.

*Martinsmyia minasensis* (Mangabeira, 1942).

Distribution: Brazil.

*Martinsmyia mollinedoi* (Le Pont and Desjeux, 1991).

Distribution: Bolivia.

*Martinsmyia oliveirai* (Martins, Silva and Falcão, 1970).

Distribution: Brazil.

*Martinsmyia pisuquia* (Ogusuku, Guevara, Revilla, Inga and Pérez, 2001).

Distribution: Peru.

*Martinsmyia quadrispinosa* (Floch and Chassignet, 1947).

Distribution: French Guiana.

*Martinsmyia reginae* Carvalho, Brazil, Sanguinette and Andrade Filho, 2010.

Distribution: Brazil.

*Martinsmyia waltoni* (Arias, Freitas and Barrett, 1984).

Distribution: Brazil.

Gasparviannai group Young and Fairchild, 1974.

*Martinsmyia cipoensis* (Martins, Falcão and Silva, 1964).

Distribution: Brazil.

*Martinsmyia gasparviannai* (Martins, Godoy and Silva, 1962).

Distribution: Brazil.

*Nyssomyia* Barretto, 1962 (20 species).

*Nyssomyia anduzei* (Rozeboom, 1942).

Distribution: Brazil, Costa Rica, French Guiana, Panama, Peru, Venezuela.

*Nyssomyia antunesi* (Coutinho, 1939).

Distribution: Bolivia, Brazil, Colombia, French Guiana, Peru, Surinam, Trinidad and Tobago, Venezuela.

*Nyssomyia bibinae* (Léger and Abonnenc, 1988).

Distribution: French Guiana.

*Nyssomyia delsionatali* Galati and Galvis, 2012.

Distribution: Brazil.

*Nyssomyia edentula* (De León, 1971).

Distribution: Costa Rica, Guatemala, Honduras, Panama.

*Nyssomyia elongata* (Floch and Abonnenc, 1945).

Distribution: French Guiana.

*Nyssomyia fraihai* Martins, Falcão and Silva, 1979.

Distribution: Bolivia, Brazil, Peru, Venezuela.

*Nyssomyia hernandezi* (Ortiz, 1965).

Distribution: Colombia, Venezuela.

*Nyssomyia intermedia* (Lutz and Neiva, 1912).

Distribution: Brazil.

*Nyssomyia neivai* (Pinto, 1926).

Distribution: Argentina, Bolivia, Brazil, Paraguay.

*Nyssomyia pajoti* (Abonnenc, Légerand Fauran 1979).

Distribution: Brazil, Colombia, French Guiana, Peru, Surinam.

*Nyssomyia richardwardi* (Ready and Fraiha, 1981).

Distribution: Bolivia, Brazil, Colombia, Ecuador, Peru.

*Nyssomyia shawi* (Fraiha, Ward and Ready, 1981).

Distribution: Bolivia, Brazil, Colombia, Peru.

*Nyssomyia sylvicola* (Floch and Abonnenc, 1945).

Distribution: Brazil, French Guiana.

*Nyssomyia trapidoi* (Fairchild and Hertig, 1952).

Distribution: Colombia, Costa Rica, Ecuador, Guatemala, Honduras, Nicaragua, Panama.

*Nyssomyia umbratilis* (Ward and Fraiha, 1977).

Distribution: Bolivia, Brazil, Colombia, French Guiana, Peru, Surinam, Venezuela.

*Nyssomyia urbinatti* Galati and Galvis, 2012.

Distribution: Brazil.

*Nyssomyia whitmani* (Antunes and Coutinho, 1939).

Distribution: Argentina, Bolivia, Brazil, French Guiana, Paraguay, Peru, Surinam.

*Nyssomyia ylephiletor* (Fairchild and Hertig, 1952).

Distribution: Belize, Colombia, Costa Rica, Ecuador, Guatemala, Honduras, Mexico, Nicaragua, Panama.

*Nyssomyia yuilli* (Young and Porter, 1972).

Distribution: Bolivia, Colombia, Ecuador, Peru, Venezuela.

*Psathyromyia* Barretto, 1962 (47 species).

(*Forattiniella*) Vargas, 1978.

*Psathyromyia* (*Forattiniella*) *abunaensis* (Martins, Falcão and Silva, 1965).

Distribution: Bolivia, Colombia, Ecuador, Brazil, Peru.

*Psathyromyia* (*Forattiniella*) *antezanai* (Le Pont, Dujardin, Mouchet and Desjeux, 1990).

Distribution: Bolivia.

*Psathyromyia* (*Forattiniella*) *aragaoi* (Costa Lima, 1932).

Distribution: Bolivia, Brazil, Colombia, Costa Rica, Ecuador, French Guiana, Panama, Paraguay, Peru, Trinidad and Tobago, Venezuela.

*Psathyromyia* (*Forattiniella*) *barrettoi barrettoi* (Mangabeira, 1942).

Distribution: Bolivia, Brazil, Colombia, Ecuador, French Guiana, Peru, Surinam, Trinidad and Tobago.

*Psathyromyia* (*Forattiniella*) *barrettoi majuscula* (Young, 1979).

Distribution: Colombia, Costa Rica, Ecuador, El Salvador, Honduras, Nicaragua, Panama.

*Psathyromyia* (*Forattiniella*) *brasiliensis* (Costa Lima, 1932).

Distribution: Brazil, French Guiana, Peru.

*Psathyromyia* (*Forattiniella*) *campograndensis* (Oliveira, Andrade-Filho, Falcão and Brazil, 2001).

Distribution: Argentina, Brazil, French Guiana.

*Psathyromyia* (*Forattiniella*) *carpenteri* (Fairchild and Hertig, 1953).

Distribution: Belize, Colombia, Costa Rica, Mexico, Panama.

*Psathyromyia* (*Forattiniella*) *castilloi* (Leon, Mollinedo and Le Pont, 2009).

Distribution: Bolivia, Ecuador, French Guiana.

*Psathyromyia* (*Forattiniella*) *coutinhoi* (Mangabeira, 1942).

Distribution: Bolivia, Brazil, Peru.

*Psathyromyia* (*Forattiniella*) *elizabethdorvalae* Brilhante, Sábio and Galati, 2017.

Distribution: Brazil.

*Psathyromyia* (*Forattiniella*) *inflata* (Floch and Abonnenc, 1944).

Distribution: Bolivia, Brazil, French Guiana.

*Psathyromyia* (*Forattiniella*) *lutziana* (Costa Lima, 1932).

Distribution: Bolivia, Brazil, Colombia, French Guiana, Paraguay, Peru, Surinam, Venezuela.

*Psathyromyia* (*Forattiniella*) *naftalekatzi* (Falcão, Andrade Filho, Almeida and Brandão-Filho, 2000).

Distribution: Brazil.

*Psathyromyia* (*Forattiniella*) *pascalei* (Coutinho and Barretto, 1940).

Distribution: Argentina, Brazil.

*Psathyromyia* (*Forattiniella*) *pradobarrientosi* (Le Pont, Matias, Martínez and Dujardin, 2004).

Distribution: Bolivia, Brazil.

*Psathyromyia* (*Forattiniella*) *runoides* (Faichild and Hertig, 1953).

Distribution: Colombia, Costa Rica, Ecuador, Panama, Peru.

*†Psathyromyia* (*Forattiniella*) *schleei* (Peñalver and Grimaldi, 2005).

Distribution: Dominican Republic.

*Psathyromyia* (*Forattiniella*) *texana* (Dampf, 1938).

Distribution: Mexico, United States.

(*Psathyromyia*) *s.s*. Galati, 1995.

Lanei series Theodor, 1965.

*Psathyromyia* (*Psathyromyia*) *digitata* (Damasceno and Arouck, 1950).

Distribution: Brazil.

*Psathyromyia* (*Psathyromyia*) *lanei* (Barretto and Coutinho, 1941).

Distribution: Argentina, Brazil, Paraguay.

*Psathyromyia* (*Psathyromyia*) *pelloni* (Sherlock and Alencar, 1959).

Distribution: Brazil.

Shannoni series Fairchild, 1955.

*Psathyromyia* (*Psathyromyia*) *abonnenci* (Floch and Chassignet, 1947).

Distribution: Bolivia, Brazil, Colombia, Ecuador, French Guiana, Panama, Peru, Surinam, Venezuela.

*Psathyromyia* (*Psathyromyia*) *baratai* Sábio, Andrade and Galati, 2015.

Distribution: Brazil.

*Psathyromyia* (*Psathyromyia*) *barretti* Alves and Freitas, 2016.

Distribution: Brazil.

*Psathyromyia* (*Psathyromyia*) *bigeniculata* (Floch and Abonnenc, 1941).

Distribution: Argentina, Brazil, French Guiana, Paraguay.

*Psathyromyia* (*Psathyromyia*) *campbelli* (Damasceno, Causey and Arouck, 1945).

Distribution: Bolivia, Brazil, Colombia, French Guiana, Peru, Venezuela.

*Psathyromyia* (*Psathyromyia*) *cratifer* (Fairchild and Hertig, 1961).

Distribution: Belize, Costa Rica, Honduras, Mexico, Panama.

*Psathyromyia* (*Psathyromyia*) *dasymera* (Fairchild and Hertig, 1961).

Distribution: Belize, Brazil, Colombia, Costa Rica, Ecuador, Mexico, Nicaragua, Panama, Venezuela.

*Psathyromyia* (*Psathyromyia*) *dendrophyla* (Mangabeira, 1942).

Distribution: Bolivia, Brazil, Colombia, Ecuador, French Guiana, Peru, Surinam, Venezuela.

*Psathyromyia* (*Psathyromyia*) *guatemalensis* (Porter and Young, 1986).

Distribution: Guatemala.

*Psathyromyia* (*Psathyromyia*) *lerayi* (Le Pont, Martinez, Torrez-Espejo and Dujardin, 1998).

Distribution: Bolivia, Colombia.

*Psathyromyia* (*Psathyromyia*) *limai* (Fonseca, 1935) .

Distribution: Brazil.

*Psathyromyia* (*Psathyromyia*) *punctigeniculata* (Floch and Abonnenc, 1944).

Distribution: Argentina, Bolivia, Brazil, Colombia, Ecuador, French Guiana, Panama, Peru, Surinam, Venzuela.

*Psathyromyia* (*Psathyromyia*) *ribeirensis* Sábio, Andrade and Galati, 2014 .

Distribution: Brazil.

*Psathyromyia* (*Psathyromyia*) *scaffi* (Damasceno and Arouck, 1956).

Distribution: Bolivia, Brazil, Colombia, French Guiana, Peru, Surinam.

*Psathyromyia* (*Psathyromyia*) *shannoni* (Dyar, 1929).

Distribution: Belize, Bolivia, Colombia, Costa Rica, Ecuador, French Guiana, Guatemala, Honduras, Mexico, Nicaragua, Panama, Peru, Surinam, Trinidad and Tobago, United States, Venezuela.

*Psathyromyia* (*Psathyromyia*) *soccula* (Fairchild and Hertig, 1961).

Distribution: Costa Rica, Panama.

*Psathyromyia* (*Psathyromyia*) *souzacastroi* (Damasceno and Causey, 1944).

Distribution: Brazil.

*Psathyromyia* (*Psathyromyia*) *undulata* (Fairchild and Hertig, 1950).

Distribution: Belize, Bolivia, Colombia, Costa Rica, Ecuador, El Salvador, French Guiana, Guatemala, Honduras, Panama.

*Psathyromyia* (*Psathyromyia*) *volcanensis* (Fairchild and Hertig, 1950).

Distribution: Bolivia, Costa Rica, Panama.

(*Xiphopsathyromyia*) Ibáñez-Bernal and Marina, 2015.

*Psathyromyia* (*Xiphopsathyromyia*) *aclydifera* (Fairchild and Hertig, 1952).

Distribution: Belize, Bolivia, Colombia, Costa Rica, Ecuador, Guatemala, Honduras, Mexico, Nicaragua, Panama.

*Psathyromyia* (*Xiphopsathyromyia*) *dreisbachi* (Causey and Damasceno, 1945).

Distribution: Bolivia, Brazil, Colombia, Ecuador, French Guiana, Peru, Surinam, Venezuela.

*Psathyromyia* (*Xiphopsathyromyia*) *hermanlenti* (Martins, Silva and Falcão, 1970).

Distribution: Brazil.

*Psathyromyia* (*Xiphopsathyromyia*) *ruparupa* (Martins, Llanos and Silva, 1976).

Distribution: Bolivia, Brazil, Peru.

*Incertae sedis*

*Psathyromyia maya* Ibáñez-Bernal, May-Uc and Rebollar-Téllez, 2010.

Distribution: Mexico.

*Psathyromyia* (*Psathyromyia*) *pifanoi* (Ortiz, 1972).

Distribution: Brazil, Colombia, Venezuela, Peru.

*Psychodopygus* Mangabeira, 1941 (39 species).

Arthuri series Barretto, 1962.

*Psychodopygus arthuri* (Fonseca, 1936).

Distribution: Brazil.

*Psychodopygus lloydi* (Antunes, 1937).

Distribution: Brazil.

*Psychodopygus matosi* (Barretto and Zago, 1956).

Distribution: Brazil.

Bispinosus series Fairchild and Hertig, 1951.

*Psychodopygus bispinosus* (Fairchild and Hertig, 1951).

Distribution: Belize, Brazil, Colombia, Costa Rica, Ecuador, French Guiana, Honduras, Guatemala, Mexico, Panama, Surinam.

Davisi series Barretto, 1962.

*Psychodopygus amazonensis* (Root, 1934).

Distribution: Bolivia, Brazil, Colombia, Ecuador, French Guiana, Peru, Surinam, Trinidad and Tobago, Venezuela.

Distribution: Bolivia, Brazil, Colombia, Peru.

Chagasi series Barretto, 1962.

*Psychodopygus bernalei* (Osorno-Mesa, Morales and Osorno, 1967).

Distribution: Bolivia, Brazil, Colombia, Venezuela.

*Psychodopygus chagasi* (Costa Lima, 1941).

Distribution: Brazil, Colombia, Peru, Venezuela.

*Psychodopygus complexus* (Mangabeira, 1941).

Distribution: Bolivia, Brazil.

*Psychodopygus douradoi* (Fé, Freitas and Barrett, 1998).

Distribution: Brazil.

*Psychodopygus fairtigi* (Martins, 1970).

Distribution: Colombia.

*Psychodopygus killicki* (Feliciangeli, Ramirez-Pérez and Ramirez, 1988).

Distribution: Venezuela.

*Psychodopygus leonidasdeanei* (Fraiha, Ryan, Ward, Lainson and Shaw, 1986).

Distribution: Brazil.

*Psychodopygus squamiventris maripaensis* (Floch and Abonnenc, 1946).

Distribution: Brazil, French Guiana, Surinam.

*Psychodopygus squamiventris squamiventris* (Lutz and Neiva, 1912).

Distribution: Brazil, Guiana, Peru, Venezuela.

*Psychodopygus wellcomei* Fraiha, Shaw and Lainson, 1971.

Distribution: Brazil, Venezuela.

*Psychodopygus claustrei* (Abonnenc, Léger and Fauran, 1979).

Distribution: Bolivia, Brazil, Colombia, French Guiana, Peru, Surinam, Venezuela.

*Psychodopygus davisi* (Root, 1934).

Distribution: Bolivia, Brazil, Colombia, Ecuador, French Guiana, Peru, Surinam, Venezuela.

*Psychodopygus parimaensis* (Ortiz and Álvarez, 1972).

Distribution: Venezuela.

Guyanensis series Barretto, 1962.

*Psychodopygus corossoniensis* (Le Pont and Pajot, 1978).

Distribution: Brazil, Costa Rica, French Guiana, Mexico, Panama, Surinam.

*Psychodopygus dorlinsis* (Le Pont and Desjeux, 1982).

Distribution: Brazil, French Guiana.

*Psychodopygus francoisleponti* Zapata, Depaquit and León,2012.

Distribution: Brazil, Ecuador.

*Psychodopygus geniculatus* (Mangabeira, 1941).

Distribution: Belize, Bolivia, Brazil, Colombia, Costa Rica, Ecuador, French Guiana, Guatemala, Panama, Peru, Nicaragua, Venezuela.

*Psychodopygus guyanensis* (Floch and Abonnenc, 1941).

Distribution: Brazil, French Guiana, Surinam.

*Psychodopygus lainsoni* (Fraiha and Ward, 1974).

Distribution: Bolivia, Brazil, Peru.

*Psychodopygus luisleoni* León, Mollinedo and Le Pont, 2009.

Distribution: Ecuador.

Panamensis series Young and Fairchild, 1974.

*Psychodopygus ayrozai* (Barretto and Coutinho, 1940).

Distribution: Bolivia, Brazil, Colombia, Ecuador, French Guiana, Panama, Peru, Trinidad and Tobago, Venezuela.

*Psychodopygus carrerai* (Barretto, 1946).

Distribution: Bolivia, Brazil, Colombia, Ecuador, Peru, Venezuela.

*Psychodopygus fairchildi* (Barretto, 1966).

Distribution: Brazil.

*Psychodopygus hirsutus* (Mangabeira, 1942).

Distribution: Bolivia, Brazil, Colombia, Ecuador, French Guiana, Peru, Surinam.

*Psychodopygus nicaraguensis* (Fairchild and Hertig, 1961).

Distribution: Brazil, Panama, Nicaragua.

*Psychodopygus joliveti* Le Pont, León, Galati and Dujardin, 2009.

Distribution: French Guiana.

*Psychodopygus llanosmartinsi* Fraiha and Ward, 1980.

Distribution: Bolivia, Brazil, Peru.

*Psychodopygus nocticolus* (Young, 1973).

Distribution: Bolivia, Colombia, Ecuador, French Guiana, Mexico, Panama, Peru.

*Psychodopygus panamensis* (Shannon, 1926).

Distribution: Belize, Brazil, Colombia, Costa Rica, Ecuador, French Guiana, Guatemala, Honduras, Mexico, Nicaragua, Panama, Peru, Surinam, Venezuela.

*Psychodopygus paraensis* (Costa Lima, 1941).

Distribution: Bolivia, Brazil, Colombia, Ecuador, French Guiana, Peru, Surinam, Venezuela.

*Psychodopygus recurvus* (Young, 1973).

Distribution: Colombia, Panama.

*Psychodopygus thula* (Young, 1979).

Distribution: Colombia, Costa Rica, Ecuador, Honduras, Panama.

*Psychodopygus yasuniensis* Leon, Neira and Le Pont, 2009.

Distribution: Ecuador.

*Psychodopygus yucumensis* (Le Pont, Caillard, Tibayrenc and Desjeux, 1986).

*Trichophoromyia* Barretto, 1962 (45 species).

*Trichophoromyia acostai* (Llanos, 1966).

Distribution: Peru.

*Trichophoromyia adelsonsouzai* Santos, Silva, Barata, Andrade and Galati, 2013.

Distribution: Brazil.

*Trichophoromyia arevaloi* Galati and Cáceres, 1999.

Distribution: Peru.

*Trichophoromyia auraensis* (Mangabeira, 1942).

Distribution: Bolivia, Brazil, Colombia, Peru, Surinam, Venezuela.

*Trichophoromyia beniensis* (Le Pont and Desjeux, 1987).

Distribution: Bolivia.

*Trichophoromyia bettinii* (Feliciangeli, Ramirez Pérez and Ramirez, 1988).

Distribution: Colombia, Venezuela.

*Trichophoromyia brachipyga* (Mangabeira, 1942).

Distribution: Brazil, French Guiana.

*Trichophoromyia castanheirai* (Damasceno, Causey and Arouck, 1945).

Distribution: Brazil.

*Trichophoromyia cellulana* (Young, 1979).

Distribution: Colombia, Ecuador, Peru.

*Trichophoromyia clitella* (Young and Pérez, 1994).

Distribution: Brazil, Peru.

*Trichophoromyia dunhami* (Causey and Damasceno, 1945).

Distribution: Brazil.

*Trichophoromyia eurypyga* (Martins, Falcão and Silva, 1963).

Distribution: Brazil, Venezuela.

*Trichophoromyia flochi* (Abonnenc and Chassignet, 1948).

Distribution: Brazil, French Guiana.

*Trichophoromyia gibba* (Young and Arias, 1994).

Distribution: Brazil.

*Trichophoromyia howardi* (Young, 1979).

Distribution: Brazil, Colombia, Peru.

*Trichophoromyia incasica* (Llanos, 1966).

Distribution: Peru.

*Trichophoromyia ininii* (Floch and Abonnenc, 1943).

Distribution: Brazil, French Guiana, Surinam.

*Trichophoromyia iorlandobaratai* Vasconcelos dos Santos, Santos Neto, Sánches-Uzcategui and Galardo, 2018.

Distribution: Brazil.

*Trichophoromyia jariensis* Cavalcante, Rodrigues and Galati, 2024.

Distribution: Brazil.

*Trichophoromyia lopesi* (Damasceno, Causey and Arouck, 1945).

Distribution: Brazil.

*Trichophoromyia loretonensis* (Llanos, 1964).

Distribution: Brazil, Peru.

*Trichophoromyia macrisae* Méndez-Cardona and Cabrera-Quintero, 2024.

Distribution: Peru.

*Trichophoromyia meirai* (Causey and Damasceno, 1945).

Distribution: Brazil.

*Trichophoromyia melloi* (Causey and Damasceno, 1945).

Distribution: Brazil.

*Trichophoromyia napoensis* (Young and Rogers, 1984).

Distribution: Ecuador.

*Trichophoromyia nautaensis* (Fernandez, Lopez, Cardenas and Requena, 2015).

Distribution: Peru.

*Trichophoromyia nemorosa* (Young and Pérez, 1994).

Distribution: Peru.

*Trichophoromyia octavioi* (Vargas, 1949).

Distribution: Bolivia, Brazil, Peru.

*Trichophoromyia omagua* (Martins, Llanos and Silva, 1976).

Distribution: Brazil, Peru.

*Trichophoromyia pabloi* (Barretto, Burbano and Young, 2002).

Distribution: Colombia, Ecuador.

*Trichophoromyia pastazaensis* (Fernandez, Carbajal, Alexander and Need, 1993).

Distribution: Peru.

*Trichophoromyia peixotoi* Rodrigues, Pinto and Galati, 2023.

Distribution: Brazil.

*Trichophoromyia readyi* (Ryan, 1986).

Distribution: Brazil.

*Trichophoromyia reburra* (Fairchild and Hertig, 1961).

Distribution: Brazil, Colombia, Costa Rica, Ecuador, Panama.

*Trichophoromyia reinerti* (Young and Duncan, 1994).

Distribution: Brazil.

*Trichophoromyia rostrans* (Summers, 1912).

Distribution: Brazil.

*Trichophoromyia ruifreitasi* Oliveira, Teles, Medeiros, Camargo and Pesso, 2015.

Distribution: Brazil.

*Trichophoromyia ruii* (Arias and Young, 1982).

Distribution: Brazil, Colombia.

*Trichophoromyia saltuosa* (Young, 1979).

Distribution: Colombia.

*Trichophoromyia sinuosa* (Young and Duncan, 1994).

Distribution: Peru.

*Trichophoromyia ubiquitalis* (Mangabeira, 1942).

Distribution: Bolivia, Brazil, Colombia, Ecuador, French Guiana, Peru, Surinam, Venezuela.

*Trichophoromyia uniniensis* Ladeia-Andrade, Fé, Sanguinette and Andrade Filho,

2014

Distribution: Brazil.

*Trichophoromyia velascoi* (Le Pont and Desjeux, 1992).

Distribution: Bolivia, Peru.

*Trichophoromyia viannamartinsi* (Sherlock and Guitton, 1970).

Distribution: Brazil.

*Trichophoromyia wilkersoni* (Young and Rogers, 1984).

Distribution: Ecuador.

*Viannamyia* Mangabeira, 1941 (4 species).

*Viannamyia caprina* (Osorno-Mesa, Morales and Osorno, 1972).

Distribution: Brazil, Colombia, Costa Rica, Honduras, Panama, Peru, Nicaragua.

*Viannamyia fariasi* (Damasceno, Causey and Arouck, 1945).

Distribution: Brazil, French Guiana.

*Viannamyia furcata* (Mangabeira, 1941).

Distribution: Bolivia, Brazil, Colombia, Costa Rica, Ecuador, French Guiana, Peru, Venezuela.

*Viannamyia tuberculata* (Mangabeira, 1941).

Distribution: Bolivia, Brazil, Colombia, French Guiana, Panama, Peru, Surinam, Venezuela.

**Eastern Hemisphere (Old World) (Total: 514 valid species)**

Tribe HERTIGIINI Abonnenc and Léger, 1976.

Subtribe IDIOPHLEBOTOMINA Galati, 1995.

*Chinius* Leng, 1987 (4 species).

*Chinius barbazani* Depaquit, Léger and Beales, 2006.

Distribution: Thailand.

*Chinius eunicegalatiae* Depaquit, Léger and Beales, 2006.

Distribution: Laos.

*Chinius junlianensis* Leng, 1987.

Distribution: China, Vietnam.

*Chinius samarensis* Léger, Depaquit and Gay, 2012.

Distribution: Philippines.

*Idiophlebotomus* Quate and Fairchild, 1961 (14 species).

*Idiophlebotomus asperulus* (Quate and Fairchild, 1961).

Distribution: Malaysia, Thailand.

*Idiophlebotomus boucheti* (Léger and Pesson, 1994).

Distribution: Indonesia.

*Idiophlebotomus dispar* Lewis, 1987.

Distribution: Indonesia.

*Idiophlebotomus erebicolus* (Quate, 1965).

Distribution: Philippines.

*Idiophlebotomus frondifer* (Lewis and Lane, 1976).

Distribution: Malaysia.

*Idiophlebotomus longiforceps* (Wang, Ku and Yuan, 1974).

Distribution: China, Laos, Thailand, Vietnam.

*Idiophlebotomus nicolegerae* Loyer, Depaquit and Gay, 2016.

Distribution: Cambodia.

*Idiophlebotomus padillarum* Léger, Depaquit and Gay, 2014.

Distribution: Philippines.

*Idiophlebotomus pholetor* (Quate and Fairchild, 1961).

Distribution: Malaysia, Philippines, Thailand.

*Idiophlebotomus sejunctus* (Quate, 1965).

Distribution: Philippines.

*Idiophlebotomus stellae* (Quate, 1965).

Distribution: Philippines.

*Idiophlebotomus teshi* (Lewis, 1978).

Distribution: Nepal, Thailand.

*Idiophlebotomus tubifer* (Lewis and Lane, 1976).

Distribution: India.

*Idiophlebotomus wellingsae* (Lewis and Dyce, 1983).

Distribution: Australia.

† *Phlebotomiella* Meunier, 1906 (1 species).

† *Phlebotomiella tipuliformis* (Meunier, 1905).

Distribution: Russia—(Kaliningrad).

† *Phlebotomites* Hennig, 1972 (6 species).

† *Phlebotomites aphoe* Stebner, Kraemer, Ibáñez-Bernal and Wagner, 2015.

Distribution: Myanmar.

† *Phlebotomites brevifilis* Hennig, 1972.

Distribution: Lebanon.

† *Phlebotomites burmaticus* Malak, Salamé and Azar, 2013.

Distribution: Myanmar.

† *Phlebotomites grimaldii* Malak, Salamé and Azar, 2013.

Distribution: Myanmar.

† *Phlebotomites longifilis* Hennig, 1972.

Distribution: Lebanon.

† *Phlebotomites neli* Malak, Salamé and Azar, 2013.

Distribution: Myanmar.

*Spelaeophlebotomus* Theodor, 1948. (2 species)

*Spelaeophlebotomus gigas* (Parrot and Schwetz, 1937).

Distribution: Angola, Cameroon, Central African Republic, Democratic Republic of Congo, Gabon, Guinea, Mali, Republic of Congo.

*Spelaeophlebotomus minteri* (Lewis, 1982).

Distribution: Tanzania.

Tribe PHLEBOTOMINI Rondani, 1840.

Subtribe AUSTRALOPHLEBOTOMINA Galati, 1995.

*Australophlebotomus* Theodor, 1948 (11 species).

*Australophlebotomus acuminatus* (Lewis and Dyce, 1982).

Distribution: Australia.

*Australophlebotomus brevilis* (Tonnoir, 1935).

Distribution: Australia.

*Australophlebotomus brevifiloides* (Fairchild, 1952).

Distribution: Australia.

*Australophlebotomus buccinator* (Fairchild, 1952).

Distribution: Australia.

*Australophlebotomus dycei* (Seccombe, Ready and Huddlestin, 1993).

Distribution: Australia.

*Australophlebotomus mackerrasi* (Lewis and Dyce, 1982).

Distribution: Australia.

*Australophlebotomus maduloae* Léger and Pesson, 1993.

Distribution: New Caledonia.

*Australophlebotomus notteghemae* Léger and Pesson, 1993.

Distribution: New Caledonia.

*Australophlebotomus papauensis* (Fairchild, 1952).

Distribution: Papua-New Guinea.

*Australophlebotomus pexopharynx* (Fairchild, 1952).

Distribution: Australia.

*Australophlebotomus trifilis* (Quate and Quate, 1967).

Distribution: Indonesia.

Subtribe PHLEBOTOMINA Rondani 1840.

*Parvidens* Quate and Fairchild, 1961 (4 species).

*Parvidens arida* (Davidson, 1982).

Distribution: Namibia, Zimbabwe.

*Parvidens heischi* (Kirk and Lewis, 1950).

Distribution: Cameroon, Ethiopia, Kenya, Mozambique, Namibia, Sudan.

*Parvidens iranicus* (Lewis and Mesghali, 1964).

Distribution: Iran.

*Parvidens lesleyae* (Lewis and Kirk, 1946).

Distribution: Ethiopia, Mauritania, Senegal, Sudan.

*Phlebotomus* Rondani and Berté, 1840 (130 species).

(*Abonnencius*) Morillas Márquez, Castillo Remiro and Ubeda Ontiveros, 1984.

*Phlebotomus* (*Abonnencius*) *fortunatarum* Ubeda Ontiveros, Morilla Márquez, Guevara Benífez, López-Román and Cutillas Barrios, 1982.

Distribution: Canary Islands (Spain).

(*Adlerius*) Nitzulescu, 1931.

*Phlebotomus* (*Adlerius*) *angustus* Artemiev, 1978.

Distribution: Afghanistan, Pakistan, Saudi Arabia, Tadzhikistan, Turkmenistan, Ukraine, Uzbekistan.

*Phlebotomus* (*Adlerius*) *arabicus* Theodor, 1953.

Distribution: Chad, Egypt, Ethiopia, Jordan, Lebanon, Saudi Arabia, Yemen.

*Phlebotomus* (*Adlerius*) *balcanicus* Theodor, 1958.

Distribution: Azerbaijan, Bosnia and Herzegovina, Bulgaria, Croatia, Georgia, Greece, Iran, Kosovo, Lebanon, North Macedonia, Montenegro, Romania, Serbia, Slovenia, Turkey, Ukraine (Crimea).

*Phlebotomus* (*Adlerius*) *brevis* Theodor and Mesghali, 1964.

Distribution: Azerbaijan, Georgia, Greece, Iran, Jordan, Lebanon, Malta, Turkey, Ukraine.

*Phlebotomus* (*Adlerius*) *chinensis* Newstead, 1916.

Distribution: China.

*Phlebotomus* (*Adlerius*) *comatus* Artemiev, 1978.

Distribution: Afghanistan, Iran, Nepal, Pakistan.

*Phlebotomus* (*Adlerius*) *creticus* Antoniou, Depaquit and Dvořák 2020.

Distribution: Greece.

*Phlebotomus* (*Adlerius*) *davidi* Artemiev, 1980.

Distribution: Egypt, Ethiopia, Saudi Arabia, Yemen.

*Phlebotomus* (*Adlerius*) fengi Leng and Zhang, 1994.

Distribution: China.

*Phlebotomus* (*Adlerius*) *halepensis* Theodor, 1958.

Distribution: Azerbaijan, Georgia, Greece, Iran, Israel/Palestine, Jordan, Malta, Syria, Turkey, Ukraine.

*Phlebotomus* (*Adlerius*) *hindustanicus* Theodor, 1958.

Distribution: Afghanistan, India, Nepal, Pakistan.

*Phlebotomus* (*Adlerius*) *kabulensis* Artemiev, 1978.

Distribution: Afghanistan, Iran.

*Phlebotomus* (*Adlerius*) *kyreniae* Theodor, 1958.

Distribution: Cyprus. Turkey.

*Phlebotomus* (*Adlerius*) *longiductus* Parrot, 1928.

Distribution: Afghanistan, Azerbaijan, Georgia, China, India, Iran, Pakistan, Romania, Ukraine (Crimea), Uzbekistan.

*Phlebotomus* (*Adlerius*) *naqbenius* Lewis and Büttiker, 1986.

Distribution: Saudi Arabia.

*Phlebotomus* (*Adlerius*) *rupester* Artemiev, 1978.

Distribution: Afghanistan.

*Phlebotomus* (*Adlerius*) *salangensis* Artemiev, 1978.

Distribution: Afghanistan, Iran.

*Phlebotomus* (*Adlerius*) *sichuanensis* Leng and Yin, 1983.

Distribution: China.

*Phlebotomus* (*Adlerius*) *simici* Nitzulescu, 1931.

Distribution: Albania, Austria, Bulgaria, Bosnia and Herzegovina, Croatia, Greece, Iran, Israel/Palestine, Jordan, Kosovo, Lebanon, Montenegro, North Macedonia, Romania, Serbia, Slovenia, Syria, Turkey.

*Phlebotomus* (*Adlerius*) *turanicus* Artemiev, 1974.

Distribution: Afghanistan, Iran, Tadzhikistan, Tajikistan, Turkmenistan, Uzbekistan.

*Phlebotomus* (*Adlerius*) *zulfagarensis* Artemiev, 1978.

Distribution: Iran, Turkmenistan.

(*Anaphlebotomus*) Theodor, 1948 (7 species).

*Phlebotomus* (*Anaphlebotomus*) *ajithii* Shah, Fathima, Jicksy, Saini, 2024.

Distribution: India.

*Phlebotomus* (*Anaphlebotomus*) *colabaensis* Young and Chalam, 1927.

Distribution: India, Pakistan.

*Phlebotomus* (*Anaphlebotomus*) *maynei* Sinton, 1930.

Distribution: India.

*Phlebotomus* (*Anaphlebotomus*) *rodhaini* Parrot, 1930.

Distribution: African Republic, Angola, Benin, Botswana, Cameroon, Republic of Congo, Democratic Republic of Congo, Ethiopia, Gambia, Ghana, Guinea, Kenya, Liberia, Mozambique, Nigeria, Senegal, South Africa, Sudan, Togo, Uganda.

*Phlebotomus* (*Anaphlebotomus*) *rousettus* Davidson, 1981.

Distribution: South Africa.

*Phlebotomus* (*Anaphlebotomus*) *shadenae* Depaquit, Pellot and Siriyasatien, 2023.

Distribution: Laos, Thailand.

*Phlebotomus* (*Anaphlebotomus*) *stantoni* Newstead, 1914.

Distribution: Cambodia, China, India, Indonesia, Laos, Malaysia, Sri Lanka, Thailand, Vietnam.

(*Artemievus*) Depaquit, 2021.

*Phlebotomus* (*Artemievus*) *alexandri* Sinton, 1928.

Distribution: Afghanistan, Algeria, Azerbaijan, Bulgaria, China, Cyprus, Djibouti, Egypt, Ethiopia, Greece, India, Iran, Iraq, Israel, Jordan, Kazakhstan, Kosovo, Kuwait, Lebanon, Libya, Mongolia, Morocco, Niger, North Macedonia, Oman, Pakistan, Romania, Saudi Arabia, Serbia, Spain, Sudan, Syria, Tunisia, Turkey, Turkmenistan, Ukraine (Crimea), United Arab, Uzbekistan, Emirates, Yemen.

(*Euphlebotomus*) Theodor, 1948.

*Phlebotomus* (*Euphlebotomus*) *argentipes* Annandale and Brunetti, 1908.

Distribution: Bangladesh, India, Indonesia, Laos, Malaysia, Myanmar, Nepal, Pakistan, Sri Lanka, Thailand, Vietnam.

*Phlebotomus* (*Euphlebotomus*) *autumnalis* Artemiev, 1980.

Distribution: Afghanistan.

*Phlebotomus* (*Euphlebotomus*) *barguesae* Depaquit, Müller and Léger, 2009.

Distribution: Laos, Thailand.

*Phlebotomus* (*Euphlebotomus*) *caudatus* Artemiev, 1978.

Distribution: Afghanistan.

*Phlebotomus* (*Euphlebotomus*) *gouldi* Lewis, 1978.

Distribution: Thailand.

*Phlebotomus* (*Euphlebotomus*) *kiangsuensis* Yao and Wu, 1938.

Distribution: Cambodia, China, Laos, Malaysia, Taiwan, Thailand, Vietnam.

*Phlebotomus* (*Euphlebotomus*) *marginatus* Annandale, 1910.

Distribution: Sri Lanka.

*Phlebotomus* (*Euphlebotomus*) *mascomai* Müller, Depaquit and Léger, 2007.

Distribution: Thailand, Vietnam.

*Phlebotomus* (*Euphlebotomus*) *mesghalii* Rashti and Nadin, 1970.

Distribution: Afghanistan, Iran.

*Phlebotomus* (*Euphlebotomus*) *nadimi* Lavadian, Jalali-Galousang and Seyedi-Rashi, 1997.

Distribution: Iran.

*Phlebotomus* (*Euphlebotomus*) *philippinensis* Manalang, 1930.

Distribution*:* China, Malaysia, Philippines.

*Phlebotomus* (*Euphlebotomus*) *tumenensis* Wang and Chang, 1963.

Distribution: China.

*Phlebotomus* (*Euphlebotomus*) *yunshengensis* Leng and Lewis, 1987.

Distribution: China, Vietnam.

(*Kasaulius*) Lewis, 1982.

*Phlebotomus* (*Kasaulius*) *newsteadi* Sinton, 1926.

Distribution: India.

(*Larroussius*) Nitzulescu, 1931.

*Phlebotomus* (*Larroussius*) *aculeatus* Lewis, Minter and Asford, 1974.

Distribution: Kenya, Ethiopia.

*Phlebotomus* (*Larroussius*) *ariasi* Tonnoir, 1921.

Distribution: Algeria, Andorra, France, Italy, Libya, Morocco, Portugal, Spain, Tunisia.

*Phlebotomus* (*Larroussius*) *ashfordi* Gebre-Michael and Lane, 1996.

Distribution: Ethiopia.

*Phlebotomus* (*Larroussius*) *burneyi* Lewis, 1967.

Distribution: India, Pakistan, Turkey.

*Phlebotomus* (*Larroussius*) *chadlii* Rioux, Juminer and Gibily, 1966.

Distribution: Algeria, Morocco, Tunisia.

*Phlebotomus* (*Larroussius*) *elgonensis* Ngoka, Madel and Mutinga, 1975.

Distribution: Ethiopia, Kenya.

*Phlebotomus* (*Larroussius*) *fantalensis* Lewis, Minter and Asford, 1974.

Distribution: Ethiopia.

*Phlebotomus* (*Larroussius*) *galileus* Theodor, 1958.

Distribution: Cyprus, Israel/Palestine, Jordan, Syria, Turkey.

*Phlebotomus* (*Larroussius*) *gibiensis* Lewis, Minter and Asford, 1974.

Distribution: Ethiopia.

*Phlebotomus* (*Larroussius*) *guggisbergi* Kirk and Lewis, 1952.

Distribution: Kenya, Tanzania, Uganda.

*Phlebotomus* (*Larroussius*) *ilami* Javadian, Jalali-Galousang and Seyedi-Rashi, 1997.

Distribution: Iran.

*Phlebotomus* (*Larroussius*) *kandelakii* Shchurenkova, 1929.

Distribution: Afghanistan, Albania, Armenia, Azerbaijan, Bulgaria, Georgia, India, Iran, Lebanon, Montenegro, Pakistan, Syria, Turkey.

*Phlebotomus* (*Larroussius*) *keshishiani* Shchurenkova, 1936.

Distribution: Afghanistan, Iran, Pakistan, Tajikistan, Uzbekistan.

*Phlebotomus* (*Larroussius*) *langeroni* Nitzulescu, 1930.

Distribution: Algeria, Egypt, Iran, Lebanon, Libya, Morocco, Spain, Tunisia. *Phlebotomus* (*Larroussius*) *lengi* Zhang, He and Ward, 1994.

Distribution: China.

*Phlebotomus* (*Larroussius*) *longicuspis* Nitzulescu, 1930.

Distribution: Algeria, Burkina Faso, Libya, Morocco, Spain, Tunisia.

*Phlebotomus* (*Larroussius*) *longipes* Parrot and Martin, 1939.

Distribution: Ethiopia, Kenya, Sudan.

*Phlebotomus* (*Larroussius*) *major* Annandale, 1910.

Distribution: India, Iran, Nepal, Pakistan, Nepal, Turkey.

*Phlebotomus* (*Larroussius*) *mariae* Rioux, Croset, Léger and Bailly-Choumara, 1974.

Distribution: Morroco.

*Phlebotomus* (*Larroussius*) *neglectus* Tonnoir, 1921.

Distribution: Albania, Austria, Bosnia and Herzegovina, Bulgaria, Croatia, Cyprus, Greece, Hungary, Iran, Israel/Palestine, Italy, Kosovo, Lebanon, Malta, Monaco, Montenegro, North Macedonia, Romania, Russia, Serbia, Slovenia, Syria, Switzerland, Turkey, Ukraine (Crimea).

*Phlebotomus* (*Larroussius*) *notus* Artemiev and Neronov, 1984.

Distribution: Afghanistan, Iran.

*Phlebotomus* (*Larroussius*) *orientalis* Parrot, 1936.

Distribution: Chad, Djibouti, Egypt, Ethiopia, Israel/Palestine, Jordan, Kenya, Lebanon, Niger, Rwanda, Saudi Arabia, South Yemen, Saudi Arabia, Sudan, Uganda, Yemen.

*Phlebotomus* (*Larroussius*) *pedifer* Lewis, Mutinga and Ashford, 1972.

Distribution: Ethiopia, Kenya, Sudan.

*Phlebotomus* (*Larroussius*) *perfiliewi* Parrot, 1930.

Distribution: Albania, Algeria, Armenia, Azerbaijan, Bosnia and Herzegovina, Bulgaria, Croatia, France, Greece, Hungary, Iran, Italy, Kosovo, Malta, Moldova, Monaco, Montenegro, Morocco, North Macedonia, Romania, Serbia, Slovenia, Tunisia, Turkey, Ukraine (Crimea).

*Phlebotomus* (*Larroussius*) *perniciosus* Newstead, 1911.

Distribution: Algeria, Andorra, Austria, Croatia, Cyprus, France, Germany, Italy, Libya, Malta, Monaco, Morocco, Portugal, Spain, Slovenia, Switzerland, Tunisia, United Kingdom (Jersey Island).

*Phlebotomus* (*Larroussius*) *smirnovi* Perfili’ev, 1941.

Distribution: Belarus, China, Iran, Kazakhstan, Turkmenistan, Ukraine, Uzbekistan.

*Phlebotomus* (*Larroussius*) *somaliensis* Abonnenc, Adam and Bailly-Choumara, 1959.

Distribution: Somalia.

*Phlebotomus* (*Larroussius*) *syriacus* Adler and Theodor, 1931.

Distribution: Armenia, Azerbaijan, Egypt, Georgia, Israel/Palestine, Jordan, Lebanon, Saudi Arabia, Syria, Ukraine,Turkey.

*Phlebotomus* (*Larroussius*) *tobbi* Adler and Theodor, 1930.

Distribution: Albania, Armenia, Azerbaijan, Bosnia and Herzegovina, Bulgaria, Croatia, Cyprus, Georgia, Greece, Iran, Iraq, Israel/Palestine, Italy, Jordan, Kosovo, Lebanon, Montenegro, North Macedonia, Serbia, Slovenia, Syria, Turkey.

*Phlebotomus* (*Larroussius*) *transcaucasicus* Perfili’ev, 1937.

Distribution: Azerbaijan, Georgia, Iraq, Iran, Israel/Palestine, Russia, Turkey. *Phlebotomus* (*Larroussius*) *wenyoni* Adler and Theodor, 1930.

Distribution: Georgia, Iran, Iraq, Turkey, Turkmenistan.

(*Legeromyia*) Rahola, Depaquit, Makanga and Paupy, 2013.

*Phlebotomus* (*Legeromyia*) *multihamatus* Rahola, Depaquit, Makanga and Paupy, 2013.

Distribution: Gabon.

(*Lewisius*) Depaquit and Vongphayloth, 2023.

*Phlebotomus* (*Lewisius*) *betisi* Lewis and Wharton, 1963.

Distribution: Malaysia, Thailand, Vietnam.

*Phlebotomus* (*Lewisius*) *breyi* Vongphayloth and Depaquit, 2023.

Distribution: Laos.

*Phlebotomus* (*Lewisius*) *sinxayarami* Vongphayloth and Depaquit, 2023.

Distribution: Laos.

(*Madaphlebotomus*) Depaquit, Léger and Randrianambinintsoa, 2015.

*Phlebotomus* (*Madaphlebotomus*) *artemievi* Randrianambinintsoa and Depaquit, 2020.

Distribution: Madagascar.

*Phlebotomus* (*Madaphlebotomus*) *berentiensis* (*Léger* and Rodhain, 1978).

Distribution:Madagascar.

*Phlebotomus* (*Madaphlebotomus*) *fertei* (Depaquit, Léger and Robert, 2002).

Distribution: Madagascar.

*Phlebotomus* (*Madaphlebotomus*) *fontenillei* (Depaquit, Léger and Robert, 2004).

Distribution: Madagascar.

*Phlebotomus* (*Madaphlebotomus*) *vaomalalae* (Randrianambinintsoa, Léger, Robert and Depaquit, 2013).

Distribution: Madagascar.

*Phlebotomus* (*Madaphlebotomus*) *vincenti* (Randrianambinintsoa and Depaquit, 2013).

Distribution: Madagascar.

(*Paraphlebotomus*) Theodor, 1948.

*Phlebotomus* (*Paraphlebotomus*) *andrejevi* Shakirzyanova, 1953.

Distribution: Afghanistan, China, Iran, Kazakhstan, Mongolia, Ukraine, Uzbekistan.

*Phlebotomus* (*Paraphlebotomus*) *caucasicus* Marzinovsky, 1917.

Distribution: Afghanistan, Armenia, Azerbaijan, China, Georgia, Iran, Kazakhstan, Turkey, Ukraine, Uzbekistan.

*Phlebotomus* (*Paraphlebotomus*) *chabaudi* Croset, Abonnenc and Rioux, 1970.

Distribution: Algeria, Libya, Morocco, Spain, Tunisia.

*Phlebotomus* (*Paraphlebotomus*) *gemetchi* Gebre-Michael and Balrew, 2003.

Distribution: Ethiopia.

*Phlebotomus* (*Paraphlebotomus*) *jacusieli* Theodor, 1947.

Distribution: Armenia, Azerbaijan, Cyprus, Georgia, Iran, Iraq, Israel/Palestine, Jordan, Lebanon, Russia, Syria, Turkey, Ukraine.

*Phlebotomus* (*Paraphlebotomus*) *kazeruni* Theodor and Mesghali, 1964.

Distribution: Afghanistan, Algeria, Egypt, Iran, Israel, Jordan, Kuwait, Lebanon, Morocco, Pakistan, Saudi Arabia, Syria, Tunisia, Turkey, United Arab Emirates, Yemen.

*Phlebotomus* (*Paraphlebotomus*) *mireillae* Killick-Kendrick, Tang, Johnson, Ngumbi and Robert, 1997.

Distribution: Ethiopia, Kenya.

*Phlebotomus* (*Paraphlebotomus*) *mongolensis* Sinton, 1928.

Distribution: Afghanistan, Azerbaijan, China, Iran, Kazakhstan, Mongolia, Syria, Ukraine, Uzbekistan.

*Phlebotomus* (*Paraphlebotomus*) *nuri* Lewis, 1967.

Distribution: Afghanistan, Iran, Pakistan, Uzbekistan.

*Phlebotomus* (*Paraphlebotomus*) *riouxi* Depaquit, Ferté and Léger, 2000.

Distribution: Algeria, Libya, Morocco, Spain, Tunisia.

*Phlebotomus* (*Paraphlebotomus*) *saevus* Parrot, 1939.

Distribution: Ethiopia, Israel/Palestine, Kenya, Oman, Saudi Arabia, Sudan, Yemen.

*Phlebotomus* (*Paraphlebotomus*) *sergenti* Parrot, 1917.

Distribution: Afghanistan, Algeria, Armenia, Azerbaidjan, Bosnia and Herzegovina, Bulgaria, Croatia, Cyprus, Djibouti, Egypt, Eritrea, Ethiopia, France, Georgia, Greece, India, Iran, Iraq, Israel/Palestine, Italy, Jordan, Kazakhstan, Kenya, Kosovo, Kuwait, Lebanon, Liberia, Libya, Mali, Malta, Montenegro, Morocco, Nepal, Niger, North Macedonia, Oman, Pakistan, Portugal, Qatar, Romania, Saudi Arabia, Serbia, Slovenia, Somalia, Spain, Syria, Tunisia, Turkey, United Arab Emirates, Uzbekistan, Yemen.

*Phlebotomus* (*Paraphlebotomus*) *similis* Perfil’ev, 1963.

Distribution: Albania, Azerbaijan, Croatia, Greece, Iran, North Macedonia, Romania, Russia, Serbia, Turkey, Ukraine (Crimea).

(*Phlebotomus*) Rondani and Berté, 1840.

*Phlebotomus* (*Phlebotomus*) *bergeroti* Parrot, 1934.

Distribution: Algeria, Burkina Faso, Chad, Djibouti, Egypt, Eritrea, Ethiopia, Iran, Israel, Jordan, Libya, Mauritania, Morocco, Niger, Oman, Pakistan, Qatar, Saudi Arabia, Senegal, Somalia, Sudan, Tunisia, United Arab Emirates, Yemen.

*Phlebotomus* (*Phlebotomus*) *duboscqi* Neveu-Lemaire, 1906.

Distribution: Burkina Faso, Cameroon, Central African Republic, Chad, Ethiopia, Gambia, Ghana, Kenya, Kuwait, Mali, Mauritania, Niger, Nigeria, Oman, Qatar, Saudi Arabia, Senegal, Sierra Sudan, Togo, United Arab Emirates, Yemen.

*Phlebotomus* (*Phlebotomus*) *papatasi* (Scopoli, 1786).

Distribution: Afghanistan, Albania, Algeria, Armenia, Azerbaijan, Bangladesh, Bosnia and Herzegovina, Bulgaria, Croatia, Cyprus, Egypt, Eritrea, Ethiopia, France, Georgia, Greece, Hungary, India, Iran, Iraq, Israel/Palestine, Italy, Jordan, Kazakhstan, Kenya, Kosovo, Kuwait, Kyrgyzstan, Lebanon, Libya, Malta, Montenegro, Morocco, Nepal, Niger, North Macedonia, Oman, Pakistan, Qatar, Romania, Russia, Saudi Arabia, Serbia, Slovenia, Somalia, Spain, Sudan, Sri Lanka, Syria, Tunisia, Turkey, Turkmenistan, United Arab Emirates, Ukraine (Crimea), Uzbekistan, Yemen.

*Phlebotomus* (*Phlebotomus*) *salehi* Mesghali, 1965.

Distribution: India, Iran, Pakistan.

*Phlebotomus* (*Phlebotomus) sundarai* Basak and Tandon, 1998.

Distribution: India.

(*Synphlebotomus*) Theodor, 1948.

*Phlebotomus* (*Synphlebotomus*) *ansarii* Lewis, 1957.

Distribution: Iran.

*Phlebotomus* (*Synphlebotomus*) *celiae* Minter, 1962.

Distribution: Ethiopia, Kenya.

*Phlebotomus* (*Synphlebotomus*) *eleanorae* Sinton, 1931.

Distribution: India, Iran.

*Phlebotomus* (*Synphlebotomus*) *grovei* Downes, 1971.

Distribution: Namibia.

*Phlebotomus* (*Synphlebotomus*) *katangensis* Bequaert and Walravens, 1930.

Distribution: Democratic Republic of Congo, Zimbabwe.

*Phlebotomus* (*Synphlebotomus*) *martini* Parrot, 1936.

Distribution: Ethiopia, Kenya, Somalia, Sudan, Uganda.

*Phlebotomus* (*Synphlebotomus*) *rossi* De Meillon and Lavoipierre, 1944.

Distribution: Namibia, South Africa, Zimbabwe.

*Phlebotomus* (*Synphlebotomus*) *saltiae* Léger, Haddad and Chaker, 1997.

Distribution: Israel/Palestine, Lebanon.

*Phlebotomus* (*Synphlebotomus*) *sikandraensis* Singer and Ipe, 2005.

Distribution: India.

*Phlebotomus* (*Synphlebotomus*) *taylori* Davidson, 1982.

Distribution:Zimbabwe.

*Phlebotomus* (*Synphlebotomus*) *vansomerenae* Heisch, Guggisberg and Teesdale, 1956.

Distribution: Ethiopia, Kenya.

(*Transphlebotomus*) Artemiev and Neronov, 1984.

*Phlebotomus* (*Transphlebotomus*) *anatolicus* Kasap, Dvorak, Depaquit, Alten, Votypka and Volf, 2015.

Distribution: Turkey.

*Phlebotomus* (*Transphlebotomus*) *canaaniticus* Adler and Theodor, 1931.

Distribution: Cyprus, Israel/Palestine, Jordan, Lebanon, Syria.

*Phlebotomus* (*Transphlebotomus*) *economidesi* Léger, Depaquit and Ferté, 2000.

Distribution: Cyprus, Israel/Palestine, Jordan, Turkey.

*Phlebotomus* (*Transphlebotomus*) *killicki* Dvorak, Votypka and Volf, 2015.

Distribution: Greece, Turkey.

*Phlebotomus* (*Transphlebotomus*) *mascittii* Grassi, 1908.

Distribution: Albania, Algeria, Austria, Belgium, Bosnia and Herzegovina, Bulgaria, Burundi, Croatia, Cyprus, France, Germany, Greece, Hungary, Italy, Kosovo, Luxembourg, Monaco, Montenegro, North Macedonia, Romania, Serbia, Slovakia, Slovenia, Spain, Switzerland, Turkey.

*Phlebotomus* (*Transphlebotomus*) *simonahalepae* Cazan, Erisoz Kasap and Mihalca, 2021.

Distribution: Romania.

*Incertae sedis*

†*Phlebotomus* khludae Kaddumi, 2005.

Distribution: Jordan.

† *Phlebotomus pungens* (Loew, 1845).

Distribution: Tanzania.

*Phlebotomus sinensis* Patton, 1926.

Distribution: China.

† *Phlebotomus succini* Stuckenberg, 1975.

Distribution: Baltic.

† *Phlebotomus vetus* Stebner, Kraemer, Ibáñez-Bernal and Wagner, 2015.

Distribution: Myanmar.

Subtribe SPELAEOMYIINA Artemiev, 1991.

*Spelaeomyia* Theodor, 1948. (4 species)

*Spelaeomyia darlingi* (Lewis and Kirk, 1954).

Distribution: Burkina Faso, Central African Republic, Mali, Sudan.

*Spelaeomyia emilii* (Vattier-Bernard, 1966).

Distribution: Burkina Faso, Central African Republic, Democratic Republic of Congo, Gabon, Republic of Congo.

*Spelaeomyia mirabilis* (Parrot and Wanson, 1939).

Distribution: Angola, Central African Republic, Democratic Republic of Congo, Gabon, Republic of Congo, Tanzania, Uganda.

*Spelaeomyia moucheti* (Vattier-Bernard and Abonnenc, 1967).

Distribution: Cameroon, Central African, Gabon, Republic of Congo.

Subtribe SERGENTOMYIINA Artemiev, 1991.

*Demeillonius* Sinton, 1933 (1 species).

*Demeillonius transvaalensis* (Sinton, 1933).

Distribution: South Africa.

*Grassomyia* Newstead, 1912 (7 species).

*Grassomyia dreyfussi* (Parrot, 1933) .

Distribution: Algeria, Djibouti, Eritrea, Ethiopia, Iran, Iraq, Jordan, Kenya, Libya, Morocco, Saudi Arabia, Somalia, Sri Lanka, Tunisia, Yemen.

*Grassomyia ghesquerei* (Parrot, 1929).

Distribution: Benin, Burkina Faso, Côte d'Ivoire, Democratic Republic of Congo, Ethiopia, Gambia, Ghana, Guinea, Liberia, Nigeria, Republic of Congo, Senegal, Somalia.

*Grassomyia indica* (Theodor, 1931).

Distribution: Afghanistan, Cambodia, China, HongKong, India, Indonesia, Iran, Laos, Malaysia, Nepal, Pakistan, Sri Lanka, Taiwan, Thailand.

*Grassomyia inermis* (Theodor, 1938).

Distribution: Angola, Benin, Botswana, Burkina Faso, Central African Republic, Chad, Ethiopia, Gambia, Ghana, Guinea, Kenya, Malawi, Nigeria, Senegal, South Africa, Sudan.

*Grassomyia madagascariensis* (Abonnenc, 1969).

Distribution: Madagascar.

*Grassomyia squamipleuris* (Newstead, 1912).

Distribution: Angola, Benin, Botswana, Burkina Faso, Central African Republic, Chad, Côte d'Ivoire, Democratic Republic of Congo, Egypt, Ethiopia, Gambia, Ghana, Guinea, Iran, Iraq, Israel, Kenya, Kuwait, Malawi, Mali, Mozambique, Nigeria, Republic of Congo, Saudi Arabia, Senegal, Sierra Leone, South Africa, Sudan, Tanzania, Togo, Uganda, Zambia.

*Grassomyia turkestanica* Theodor and Mesghali, 1964.

Distribution: Afghanistan, Iran, Turkmenistan.

*Sergentomyia* França and Parrot, 1920 (326 species).

(*Capensomyia*) Davidson, 1979.

*Sergentomyia* (*Capensomyia*) *caffrarica* (De Meillon and Lavoipierre, 1944).

Distribution: South Africa.

*Sergentomyia* (*Capensomyia*) *capensis* (De Meillon, 1955).

Distribution: South Africa.

*Sergentomyia* (*Capensomyia*) *drakenbergi* (Davidson, 1979).

Distribution: South Africa.

*Sergentomyia* (*Capensomyia*) *haeselbarthi* (Abonnenc, 1967).

Distribution: South Africa.

*Sergentomyia* (*Capensomyia*) *kalaharia* Davidson, 1979.

Distribution: Namibia, South Africa.

*Sergentomyia* (*Capensomyia*) *luteola* Davidson, 1983.

Distribution: South Africa.

*Sergentomyia* (*Capensomyia*) *meeseri* (de Meillon and Hardy, 1953).

Distribution: South Africa, Uganda, Zimbabwe.

*Sergentomyia* (*Capensomyia*) *nama* Davidson, 1983.

Distribution: Namibia, South Africa.

*Sergentomyia* (*Capensomyia*) *namibensis* (de Meillon and Hardy, 1953).

Distribution: Namibia, South Africa.

*Sergentomyia* (*Capensomyia*) *xera* Davidson, 1979.

Distribution: Namibia, South Africa.

(*Neophlebotomus*) França and Parrot, 1920.

*Sergentomyia* (*Neophlebotomus*) *ashwanii* Saini, Shah, Jessu, T, Anns, Amju, 2024.

Distribution: India.

*Sergentomyia* (*Neophlebotomus*) *banerjii* Bej and Manna, 1996.

Distribution: India.

*Sergentomyia* (*Neophlebotomus*) *hodgsoni* (Sinton, 1933).

Distribution: Afghanistan, India, Iran, Malaysia, Pakistan, Thailand.

*Sergentomyia* (*Neophlebotomus*) *hoogstraali* (Fairchild, 1952).

Distribution: Australia, Indonesia, Papua New Guinea.

*Sergentomyia* (*Neophlebotomus*) *hunanensis* Leng, Li, Zhang, Li and Yao, 1985.

Distribution: China.

*Sergentomyia* (*Neophlebotomus*) *kottamala K*aul, 1993.

Distribution: India.

*Sergentomyia* (*Neophlebotomus*) *kurandamallai* Kaul, 1993.

Distribution: India.

*Sergentomyia* (*Neophlebotomus*) *lushanensis* Leng, Li, Zhang, Li and Yao, 1985.

Distribution: China.

*Sergentomyia* (*Neophlebotomus*) *monticola* Srinivasan, Jambulingam and Kumar 2014.

Distribution: India.

*Sergentomyia* (*Neophlebotomus*) *nilamburensis* Kaul and Prabha, 1993.

Distribution: India.

*Sergentomyia* (*Neophlebotomus*) *sonyae* Lewis, 1982.

Distribution: Oman, Saudi Arabia.

*Sergentomyia* (*Neophlebotomus*) *sumatrae* Lewis, 1987.

Distribution: Indonesia.

*Sergentomyia* (*Neophlebotomus*) *suni* Wu, 1954.

Distribution: China.

*Sergentomyia* (*Neophlebotomus*) *tambori* Lewis and Jeffery, 1978.

Distribution: Malaysia.

*Sergentomyia* (*Neophlebotomus*) *verghesei* Kaul, 1993.

Distribution: India.

*Sergentomyia* (*Neophlebotomus*) *whartoni* Lewis, 1957.

Distribution: Bangladesh, Cambodia, India, Indonesia, Laos, Malaysia, Myanmar, Thailand, Vietnam.

(*Parrotomyia*) Theodor, 1958.

*Sergentomyia* (*Parrotomyia*) *africana* (Newstead, 1912).

Distribution: Benin, Burkina Faso, Cameroon, Central African Republic, Côte d'Ivoire, Democratic Republic of Congo, Djibouti, Ethiopia, Gambia, Ghana, Guinea, Iran, Kenya, Liberia, Mali, Mauritania, Niger, Nigeria, Republic of Congo, Senegal, Sierra Leone, South Africa, Sudan, Togo, Uganda, Yemen, Zambia.

*Sergentomyia* (*Parromyia*) *asiatica* (Theodor, 1933).

Distribution: India, Israel/Palestine, Jordan, Morocco, Pakistan, Saudi Arabia.

*Sergentomyia* (*Parrotomyia*) *babu* (Annandale, 1910).

Distribution: Afghanistan, Bangladesh, India, Mauritius, Pakistan.

*Sergentomyia* (*Parrotomyia*) *baghdadis* (Adler and Theodor, 1929).

Distribution: Afghanistan, India, Iran, Iraq, Oman, Pakistan, Sri Lanka.

*Sergentomyia* (*Parromyia*) *barraudi* (Sinton, 1929) .

Distribution: Bangladesh, Cambodia, China, Hong Kong, India, Indonesia, Laos, Myanmar, Malaysia, Taiwan, Thailand, Vietnam.

*Sergentomyia* (*Parrotomyia*) *bigtii* (Manalang, 1931).

Distribution: Philippines.

*Sergentomyia* (*Parrotomyia*) *brevinervis* (Quate and Faichild, 1961).

Distribution: Malaysia.

*Sergentomyia* (*Parrotomyia*) *buruensis* Lewis and Dyce, 1989.

Distribution: Indonesia.

*Sergentomyia* (*Parrotomyia*) *clara* Lewis and Dyce, 1989.

Distribution: Australia.

*Sergentomyia* (*Parrotomyia*) *coronata* (Quate and Quate, 1967).

Distribution: Indonesia, Papua New Guinea.

*Sergentomyia* (*Parrotomyia*) *crosarai* (Parrot and Wanson, 1946).

Distribution: Angola, Democratic Republic of Congo, Liberia, Republic of Congo.

*Sergentomyia* (*Parrotomyia*) *crypta* (Quate and Quate, 1967).

Distribution: Indonesia, Papua New Guinea.

*Sergentomyia* (*Parrotomyia*) *curvata* Lewis and Dyce, 1983.

Distribution: Australia.

*Sergentomyia* (*Parrotomyia*) *dayapensis* (Manalang, 1931).

Distribution: Philippines.

*Sergentomyia* (*Parrotomyia*) *denticulata* (Quate and Fairchild, 1961).

Distribution: Malaysia.

*Sergentomyia* (*Parrotomyia*) *dolichobyssa* (Fairchild, 1952).

Distribution: Indonesia, Papua New Guinea.

*Sergentomyia* (*Parrotomyia*) *englishi* (Tonnoir, 1935).

Distribution: Australia, Papua New Guinea.

*Sergentomyia* (*Parrotomyia*) *eremitis* (Parrot and Bouquet de Jolinière, 1945).

Distribution: Algeria, Liberia, Sudan, Togo.

*Sergentomyia* (*Parrotomyia*) *freetownensis freetownensis* (Sinton, 1930).

Distribution: Angola, Benin, Central African Republic, Côte d'Ivoire, Ethiopia, Eritrea, Democratic Republic of Congo, Guinea, Republic of Congo, Guinea, Kenya, Mauritania, Senegal, Sierra Leone, South Africa, Sudan, Zimbabwe, Saudi Arabia.

*Sergentomyia* (*Parrotomyia*) *freetownensis furana* (Lewis and Kirk, 1958).

Distribution: Sudan.

*Sergentomyia* (*Parrotomyia*) *gobica* Artemiev, 1984.

Distribution: Mongolia.

*Sergentomyia* (*Parrotomyia*) *grekovi* (Khodukin, 1929).

Distribution: Afghanistan, India, Iran, Kazakhstan, Pakistan, Romania, Sri Lanka, Tajikistan, Turkey, Turkmenistan, Ukraine, Uzbekistan.

*Sergentomyia* (*Parrotomyia*) *heiseri* (Manalang, 1930).

Distribution: Philippines.

*Sergentomyia* (*Parrotomyia*) *himalayensis* (Annandale, 1910).

Distribution: India.

*Sergentomyia* (*Parrotomyia*) *insularis* (Theodor, 1938).

Distribution: India, Sri Lanka.

*Sergentomyia* (*Parrotomyia*) *jerighatiansis* Srinivasan and Jambulingam, 2013.

Distribution: India.

*Sergentomyia* (*Parrotomyia*) *kauli* Lewis, 1978.

Distribution: India.

*Sergentomyia* (*Parrotomyia*) *kebarica* (Quate and Quate, 1967).

Distribution: Indonesia.

*Sergentomyia* (*Parrotomyia*) *kwangsiensis* Yao and Wu, 1941.

Distribution: Bangladesh, Myanmar, Cambodia, China, Hong Kong, India, Indonesia, Laos, Malaysia, Taiwan, Thailand, Vietnam.

*Sergentomyia* (*Parrotomyia*) *lagunensis* (Quate, 1965).

Distribution: Philippines.

*Sergentomyia* (*Parrotomyia*) *magna* (Sinton, 1932).

Distribution: Angola, Burkina Faso, Cameroon, Central African Republic, Côte d'Ivoire, Democratic Republic of Congo, Ethiopia, Gambia, Ghana, Guinea, Mali, Mauritania, Nigeria, Saudi Arabia, Senegal, South Africa, Sudan, Togo, Zimbabwe. *Sergentomyia* (*Parrotomyia*) *majumdari* Bej and Manna, 1996.

Distribution: India.

*Sergentomyia* (*Parrotomyia*) *malabarica* (Annandale, 1910).

Distribution: India.

*Sergentomyia* (*Parrotomyia*) *mangana* (Manalang, 1930).

Distribution: Philippines.

*Sergentomyia* (*Parrotomyia*) *meridionalis* (Zielke, 1971).

Distribution: South Africa.

*Sergentomyia* (*Parrotomyia*) *modii* Lewis, 1978.

Distribution: India, Sri Lanka.

*Sergentomyia* (*Parrotomyia*) *montana* (Sinton, 1924).

Distribution: India, Nepal, Pakistan.

*Sergentomyia* (*Parrotomyia*) *moresbyi* (Fairchild, 1952).

Distribution: Australia, Papua New Guinea.

*Sergentomyia* (*Parrotomyia*) *palestinensis* (Adler and Theodor, 1927).

Distribution: Algeria, Azerbaijan, Egypt, Ethiopia, India, Iran, Iraq, Israel/Palestine Jordan, Libya, Morocco, Pakistan, Saudi Arabia, Sudan, Tunisia.

*Sergentomyia* (*Parrotomyia*) *queenslandi* (Hill, 1923).

Distribution: Australia, Indonesia, Papua New Guinea.

*Sergentomyia* (*Parrotomyia*) *rectangulata* Renganathan and Purushothaman, 2010.

Distribution: India.

*Sergentomyia* (*Parrotomyia*) *rhodesiensis* Abonnenc, 1967.

Distribution: Zimbabwe.

*Sergentomyia* (*Parrotomyia*) *rudnicki* Lewis, 1978.

Distribution: Indonesia, Malaysia, Sri Lanka.

*Sergentomyia* (*Parrotomyia*) *sansaporensis* (Faichild, 1952).

Distribution: Indonesia.

*Sergentomyia* (*Parrotomyia*) *santokhi* Singh and Ipe, 2005.

Distribution: India.

*Sergentomyia* (*Parrotomyia*) *shorttii* (Adler and Theodor, 1927).

Distribution: Bangladesh, India, Pakistan, Myanmar.

*Sergentomyia* (*Parrotomyia*) *siamensis* (Causey, 1938).

Distribution: Laos, Thailand.

*Sergentomyia* (*Parrotomyia*) *sogdiana* (Parrot, 1928).

Distribution: Afghanistan, Iran, Tajikistan, Ukraine, Uzbekistan.

*Sergentomyia* (*Parrotomyia*) *spinifaucis* (Quate, 1965).

Distribution: Philippines.

*Sergentomyia* (*Parrotomyia*) *spinosior* (Quate and Quate, 1967).

Distribution: Indonesia.

*Sergentomyia* (*Parrotomyia*) *sumbarica* (Perfil’ev, 1933).

Distribution: Afghanistan, China, Iran, Iraq, Turkmenistan, Ukraine, Uzbekistan.

*Sergentomyia* (*Parrotomyia*) *thapari* Mitra and Roy, 1952.

Distribution: India.

*Sergentomyia* (*Parrotomyia*) *timorica* Lewis and Dyce, 1976.

Distribution: Indonesia.

*Sergentomyia* (*Parrotomyia*) *torrechantei* (Manalang, 1931).

Distribution: Philippines.

*Sergentomyia* (*Parrotomyia*) *trezkinni* Lewis and Dyce, 1983.

Distribution: Australia.

*Sergentomyia* (*Parrotomyia*) *vadhanurensis* Srinivasan and Jambulingam, 2011.

Distribution: India.

*Sergentomyia* (*Parrotomyia*) *vernoni* Lewis, 1987.

Distribution: Indonesia.

*Sergentomyia* (*Parrotomyia*) *yercaudensis* Ilango, 2004.

Distribution: India.

*Sergentomyia* (*Parrotomyia*) *yini* Leng and Lin, 1991.

Distribution: China.

*Sergentomyia* (*Parrotomyia*) *yvonnae* (Parrot and Schwetz, 1937).

Distribution: Democratic Republic of Congo.

(*Ranavalonomyia*) Depaquit, Blavier and Laroche, 2019.

*Sergentomyia* (*Ranavalonomyia*) *maroantsetraensis* Randrianambinintsoa and Depaquit, 2020.

Distribution: Madagascar.

*Sergentomyia* (*Ranavalonomyia*) *volfi* Depaquit, Blavier and Laroche, 2019.

Distribution: Madagascar.

(*Riouxomyia*) Depaquit, Blavier and Randrianambinintsoa, 2019.

*Sergentomyia* (*Riouxomyia*) *kaltenbachi* Depaquit, Blavier and Randrianambinintsoa, 2019.

Distribution: Madagascar.

(*Rondanomyia*) Theodor 1958.

*Sergentomyia* (*Rondanomyia*) *angolensis* (Abonnenc, 1968).

Distribution: Angola, Republic of Congo.

*Sergentomyia* (*Rondanomyia*) *anhuiensis* Wangon, 1991.

Distribution: China.

*Sergentomyia* (*Rondanomyia*) *arboris* (Sinton, 1931).

Distribution: India, Sri Lanka.

*Sergentomyia* (*Rondanomyia*) *balica* Lewis, 1976.

Distribution: Indonesia.

*Sergentomyia* (*Rondanomyia*) *chakravarti* (Mitra, 1953).

Distribution: India.

*Sergentomyia* (*Rondanomyia*) *collarti* (Adler, Theodor and Parrot, 1929).

Distribution: Angola, Cameroon, Central African Republic, Côte d'Ivoire, Democratic Republic of Congo, Gambia, Ghana, Guinea, Nigeria, Republic of Congo, Sierra, Sudan, Tanzania, Uganda.

*Sergentomyia* (*Rondanomyia*) *corneti* Pastre, 1975.

Distribution: Senegal.

*Sergentomyia* (*Rondanomyia*) *decipiens* (Theodor, 1931).

Distribution: Cameroon, Central African Republic, Democratic Republic of Congo, Côte d'Ivoire, Guinea, Kenya, Nigeria, Republic of Congo, Senegal, Sudan, Tanzania, Uganda.

*Sergentomyia* (*Rondanomyia*) *dhandai* Lewis, 1978.

Distribution: India.

*Sergentomyia* (*Rondanomyia*) *dolichopa* (Abonnenc and Courtois, 1970).

Distribution: Djibouti, Iran, Yemen.

*Sergentomyia* (*Rondanomyia*) *dureni* (Parrot, 1934).

Distribution: Angola, Burkina Faso, Central African Republic, Chad, Côte d'Ivoire, Democratic Republic of Congo, Ethiopia, Gambia, Ghana, Guinea, Kenya, Liberia, Nigeria, Republic of Congo, Senegal, Sudan, Swaziland, Tanzania.

*Sergentomyia* (*Rondanomyia*) *dyemkoumai* (Abonnenc, 1964).

Distribution: Côte d'Ivoire, Republic of Congo.

*Sergentomyia* (*Rondanomyia*) *gemmea* Lewis and Jeffery, 1978.

Distribution: Laos, Malaysia, Thailand.

*Sergentomyia* (*Rondanomyia*) *gombaki* (Lewis and Wharton, 1963).

Distribution: Malaysia, Thailand.

*Sergentomyia* (*Rondanomyia*) *goodmani comorensis* Depaquit, Randrianambinintsoa and Léger, 2012.

Distribution: Union of the Comoros.

*Sergentomyia* (*Rondanomyia*) *goodmani goodmani* Depaquit and Robert, 2005.

Distribution: Madagascar.

*Sergentomyia* (*Rondanomyia*) *grejbinei* (Vattier-Bernard, 1971).

Distribution: Democratic Republic of Congo, Republic of Congo.

*Sergentomyia* (*Rondanomyia*) *grilloti* (Vattier-Bernard and Bimangou, 1975).

Distribution: Republic of Congo.

*Sergentomyia* (*Rondanomyia*) *hamidi* Lewis and Jeffery, 1978.

Distribution: Malaysia.

*Sergentomyia* (*Rondanomyia*) *harveyi* (Heisch, Guggisberg and Teesdale, 1956).

Distribution: Ethiopia, Kenya.

*Sergentomyia* (*Rondanomyia*) *hitchensi* (Manalang, 1930).

Distribution: Philippines.

*Sergentomyia* (*Rondanomyia*) *ingrami* (Newstead, 1914).

Distribution: Angola, Benin, Burkina Faso, Cameroon, Central African Republic, Côte d'Ivoire, Democratic Republic of Congo, Ethiopia, Gambia, Ghana, Guinea, Kenya, Liberia, Nigeria, Republic of Congo, Senegal, Sudan, Togo, Uganda.

*Sergentomyia* (*Rondanomyia*) *iyengari* (Sinton, 1933) s.s.

Distribution: India.

*Sergentomyia* (*Rondanomyia*)* iyengari* (Sinton, 1933) s. l.

Distribution: Cambodia, China, India, Laos, Malaysia, Taiwan, Thailand, Vietnam.

*Sergentomyia* (*Rondanomyia*) *jefferyi* Lewis, 1978.

Distribution: Malaysia.

*Sergentomyia* (*Rondanomyia*) *kelantani* (Lewis and Wharton, 1963).

Distribution: Malaysia.

*Sergentomyia* (*Rondanomyia*) *khawi* Raynal, 1936.

Distribution: Cambodia, China, Laos, Thailand.

*Sergentomyia* (*Rondanomyia*) *kirki* (Parrot, 1948).

Distribution: Kenya, Malawi, Sudan.

*Sergentomyia* (*Rondanomyia*) *kitonyii* (Minter, 1963).

Distribution: Ethiopia, Kenya.

*Sergentomyia* (*Rondanomyia*) *koloshanensis* (Yao, 1946).

Distribution: China.

*Sergentomyia* (*Rondanomyia*) *kueichenae* Leng and He, 1995.

Distribution: China.

*Sergentomyia* (*Rondanomyia*) *linearis* Lewis, 1978.

Distribution: India, Malaysia.

*Sergentomyia* (*Rondanomyia*) *machadoi* (Abonnenc, 1968).

Distribution: Angola.

*Sergentomyia* (*Rondanomyia*) *macintoshi* (Abonnenc and Pastre, 1972).

Distribution: South Africa.

*Sergentomyia* (*Rondanomyia*) *malayae* Lewis, 1957.

Distribution: Malaysia, Sri Lanka.

*Sergentomyia* (*Rondanomyia*) *nankingensis* (Ho and Wu, 1964).

Distribution: China.

*Sergentomyia* (*Rondanomyia*) *notata* (Parrot, 1938).

Distribution: Ethiopia.

*Sergentomyia* (*Rondanomyia*) *ozbeli* Depaquit and Randrianambinintsoa, 2019.

Distribution: Madagascar.

*Sergentomyia* (*Rondanomyia*) *pawlowski* (Perfil'ev, 1933).

Distribution: Afghanistan, Armenia, Iran, Iraq, Turkey, Turkmenistan, Ukraine, Uzbekistan.

*Sergentomyia* (*Rondanomyia*) *perturbans* (de Meijere, 1909).

Distribution: Cambodia, Indonesia, Laos, Malaysia, Thailand, Vietnam.

*Sergentomyia* (*Rondanomyia*) *purii* (Sinton, 1931).

Distribution: India.

*Sergentomyia* (*Rondanomyia*) *quanzhouensis* Leng, 1987.

Distribution: China.

*Sergentomyia* (*Rondanomyia*) *quatei* Lewis, 1978.

Distribution: Malaysia, Thailand.

*Sergentomyia* (*Rondanomyia*) *serrata* (Parrot and Malbrant, 1945).

Distribution: Democratic Republic of Congo, Ethiopia, Kenya, Republic of Congo, Sudan, Uganda.

*Sergentomyia* (*Rondanomyia*) *squamirostris* (Newstead, 1923).

Distribution: China, Japan.

*Sergentomyia* (*Rondanomyia*) *sylvatica* (Raynal, 1935).

Distribution: Cambodia, Laos, Malaysia, Thailand, Vietnam.

*Sergentomyia* (*Rondanomyia*) *sylvestris* (Sinton, 1924).

Distribution: India.

*Sergentomyia* (*Rondanomyia*) *teesdalei* (Minter, 1963).

Distribution: Kenya.

*Sergentomyia* (*Rondanomyia*) *tonkinensis* (Raynal, 1935).

Distribution: Vietnam.

*Sergentomyia* (*Rondanomyia*) *trouilleti* Vattier-Bernard, 1976.

Distribution: Republic of Congo.

*Sergentomyia* (*Rondanomyia*) *wuyishanensis* Leng, 1987.

Distribution: China.

*Sergentomyia* (*Rondanomyia*) *yaoi* Theodor, 1958.

Distribution: China.

*Sergentomyia* (*Rondanomyia*) *zeylanica* (Annandale, 1910).

Distribution: India, Sri Lanka.

*Sergentomyia* (*Rondanomyia*) *zhengjiani* Leng, 1983.

Distribution: China.

*Sergentomyia* (*Rondanomyia*) *zhongi* Wango, 1991.

Distribution: China.

(*Sergentomyia*) França and Parrot, 1920.

*Sergentomyia* (*Sergentomyia*) *afghanica* Artemiev, 1974.

Distribution: Afghanistan.

*Sergentomyia* (*Sergentomyia*) *agdamica* Artemiev, 1982.

Distribution: Azerbaijan.

*Sergentomyia* (*Sergentomyia*) *antennata* (Newstead, 1912).

Distribution: Algeria, Benin, Burkina Faso, Cameroon, Central African Republic, Chad, Côte d'Ivoire, Democratic Republic of Congo, Djibouti, Egypt, Ethiopia, Gabon, Gambia, Ghana, Guinea, Iran, Iraq, Israel, Jordan, Kenya, Kuwait, Lebanon, Liberia, Malawi, Mali, Mauritania, Morocco, Nigeria, Saudi Arabia, Senegal, Sierra, Somalia, South Africa, Sudan, Tanzania, Togo, Tunisia, Turkey, United Arab Emirates, Uganda, Yemen.

*Sergentomyia* (*Sergentomyia*) *arpaklensis* (Perfil'ev, 1933).

Distribution: Turkmenistan, Uzbekistan.

*Sergentomyia* (*Sergentomyia*) *ashfordi* Davidson, 1987.

Distribution: Ethiopia.

*Sergentomyia* (*Sergentomyia*) *azizi* (Adler, 1946).

Distribution: Cyprus.

*Sergentomyia* (*Sergentomyia*) *bedfordi* (Newstead, 1914).

Distribution: South Africa.

*Sergentomyia (Sergentomyia) bereiri* (Kirk and Lewis, 1952).

Distribution: Burkina Faso, Chad, Central African Republic, Côte d'Ivoire, Guinea, Nigeria, Republic of Congo, Sudan.

*Sergentomyia (Sergentomyia) bergerardi* Trouillet and Vattier-Bernard, 1978.

Distribution: Republic of Congo.

*Sergentomyia* (*Sergentomyia*) *bimangoui* Davidson, 1990.

Distribution: Benin, Burkina Faso, Chad, Gambia, Ghana, Mali, Nigeria, Senegal, Republic of Congo.

*Sergentomyia* (*Sergentomyia*) *blossi* (Kirk and Lewis, 1952).

Distribution: Benin, Burkina Faso, Chad, Gambia, Ghana, Mali, Nigeria, Senegal, Kenya.

*Sergentomyia* (*Sergentomyia*) *buxtoni* (Theodor, 1933).

Distribution: Benin, Burkina Faso, Chad, Côte d'Ivoire, Gambia, Ghana, Ethiopia, Mali, Niger, Nigeria, Senegal.

*Sergentomyia* (*Sergentomyia*) *caliginosa* Davidson, 1987.

Distribution: Malawi, Mozambique, South Africa, Zambia, Zimbabwe.

*Sergentomyia* (*Sergentomyia*) *cincta* (Parrot and Martin, 1944).

Distribution: Algeria, Central African Republic, Djibouti, Egypt, Ethiopia, Ghana, Kenya, Sudan, Uganda.

*Sergentomyia* (*Sergentomyia*) *congolensis* (Bequaert and Walravens, 1930).

Distribution: Angola, Cameroon, Democratic Republic of Congo, Eritrea, Ethiopia, Kenya, Malawi, Mozambique, Namibia, Senegal, Sudan, Swaziland, Uganda, Zambia, Zimbabwe.

*Sergentomyia* (*Sergentomyia*) *coronula* Tateng, Dondji and Krüger, 2019.

Distribution: Cameroon.

*Sergentomyia* (*Sergentomyia*) *cypriotica* (Adler, 1946).

Distribution: Cyprus.

*Sergentomyia* (*Sergentomyia*) *davidsoni* Seccombe, Ready and Huddleston, 1993.

Distribution: Eritrea, Ethiopia, Sudan.

*Sergentomyia* (*Sergentomyia*) *dentata* (Sinton, 1933).

Distribution: Afghanistan, Albania, Azerbaijan, Bosnia and Herzegovina, Bulgaria, Croatia, Cyprus, Egypt, Georgia, Greece, India, Iran, Iraq, Israel/Palestine, Jordan, Kosovo, Lebanon, Montenegro, North Macedonia, Pakistan, Saudi Arabia, Serbia, Slovenia, Sri Lanka, Syria, Turkey, Turkmenistan, Ukraine (Crimea), United Arab Emirates, Yemen.

*Sergentomyia* (*Sergentomyia*) *dissimillima* (Abonnenc, 1972).

Distribution: Burkina Faso, Côte d'Ivoire, Guinea, Republic of Congo.

*Sergentomyia* (*Sergentomyia*) *distincta* (Theodor, 1933).

Distribution: Benin, Burkina Faso, Cameroon, Central African Republic, Chad, Democratic Republic of Congo, Côte d'Ivoire, Ethiopia, Gambia, Ghana, Guinea, Kenya, Liberia, Mali, Mozambique, Namibia, Nigeria, Republic of Congo, Senegal, Sierra Leone, Sudan, Tanzania, Togo, Uganda, Zimbabwe.

*Sergentomyia* (*Sergentomyia*) *dubia* (Parrot, Mornet and Cadenat, 1934).

Distribution: Burkina Faso, Cameroon, Côte d'Ivoire, Ethiopia, Gambia, Ghana, Guinea, Mali, Mauritania, Nigeria, Republic of Congo, Senegal.

*Sergentomyia* (*Sergentomyia*) *fallax* (Parrot, 1921).

Distribution: Afghanistan, Algeria, Burkina Faso, Canary Islands, Cyprus, Djibouti, Egypt, Ethiopia, Iraq, Israel, Jordan, Lebanon, Libya, Morocco, Niger, Oman, Saudi Arabia, Yemen, Syria, Tunisia, Turkey, United Arab Emirates, Yemen.

*Sergentomyia* (*Sergentomyia*) *firmata* (Parrot and Malbrant, 1945).

Distribution: Republic of Congo.

*Sergentomyia* (*Sergentomyia*) *formica* Davidson, 1987.

Distribution: Namibia, South Africa, Zimbabwe.

*Sergentomyia* (*Sergentomyia*) *fupingensis* (Wu, 1954).

Distribution: China.

*Sergentomyia* (*Sergentomyia*) *garnhami* (Heisch, Guggisberg and Teesdale, 1956).

Distribution: Kenya, Tanzania.

*Sergentomyia* (*Sergentomyia*) *gracilis* (Kirk and Lewis, 1952).

Distribution: Kenya.

*Sergentomyia* (*Sergentomyia*) *hamoni* (Abonnenc, 1958).

Distribution: Angola, Burkina Faso, Cameroon, Central African Republic, Côte d'Ivoire, Democratic Republic of Congo, Kenya, Mali, Republic of Congo, Senegal, Sudan.

*Sergentomyia* (*Sergentomyia*) *imihra* Benallal, Depaquit and Dvořák, 2024.

Distribution: Algeria.

*Sergentomyia* (*Sergentomyia*) *impudica* (Abonnenc, 1968).

Distribution: Angola, Liberia, Republic of Congo, Uganda.

*Sergentomyia* (*Sergentomyia*) *leponti* Vattier-Bernard, 1973.

Distribution: Republic of Congo.

*Sergentomyia* (*Sergentomyia*) *logonensis* (Rageau, 1951).

Distribution: Cameroon, Central African Republic.

*Sergentomyia* (*Sergentomyia*) *lumsdeni* (Kirk and Lewis, 1950).

Distribution: Cameroon, Republic of Congo, Uganda.

*Sergentomyia* (*Sergentomyia*) *magnidentata* Davidson, 1987.

Distribution: Kenya, Liberia.

*Sergentomyia* (*Sergentomyia*) *mervynae* (Pringle, 1953).

Distribution: Afghanistan, Iran, Pakistan.

*Sergentomyia* (*Sergentomyia*) *minuta* (Rondani, 1843).

Distribution: Albania, Algeria, Bosnia and Herzegovina, Bulgaria, Canary Islands, Croatia, Cyprus, Egypt, Ethiopia, France, Greece, Israel, Italy, Jordan, Kosovo, Lebanon, Libya, Malta, Montenegro, Morocco, North Macedonia, Portugal, Romania, Serbia, Slovenia, Spain, Syria, Tunisia, Turkey, Ukraine.

*Sergentomyia* (*Sergentomyia*) *minuta parroti* (Adler and Theodor, 1927).

Distribution: Algeria, Cyprus, Greece, Libya, Morocco, Philippines, Spain, Syria, Tunisia.

*Sergentomyia* (*Sergentomyia*) *moreli* (Abonnenc and Hamon, 1958).

Distribution: Côte d'Ivoire, Liberia, Republic of Congo, Uganda.

*Sergentomyia* (*Sergentomyia*) *multidens* (Heisch, Guggisberg and Teesdale, 1956).

Distribution: Djibouti, Ethiopia, Kenya, Yemen.

*Sergentomyia* (*Sergentomyia*) *murgabiensis* (Perfil'ev, 1939).

Distribution: Afghanistan, China, Iran, Kazakhstan, Mongolia, Turkmenistan.

*Sergentomyia* (*Sergentomyia*) *nocens* (Parrot, 1951).

Distribution: Kenya, Sudan.

*Sergentomyia* (*Sergentomyia*) *ovazzai* (Pastre, 1973).

Distribution: Guinea.

*Sergentomyia* (*Sergentomyia*) *pashtunica* Artemiev, 1974.

Distribution: Afghanistan, India, Iran, Pakistan.

*Sergentomyia* (*Sergentomyia*) *pastoriana* (Parrot, Mornet and Cadenat, 1945).

Distribution: Ethiopia, Ghana, Guinea, Côte d'Ivoire, Nigeria, Sierra Leone, Sudan, Uganda.

*Sergentomyia* (*Sergentomyia*) *phadangensis* Polseela, Depaquit and Apiwathnasorn, 2016.

Distribution: Thailand.

*Sergentomyia* (*Sergentomyia*) *pondicherriensis* Srinivasan and Jambulingam, 2010.

Distribution: India, Sri Lanka.

*Sergentomyia* (*Sergentomyia*) *punjabensis* (Sinton, 1933).

Distribution: India, Pakistan, Sri Lanka.

*Sergentomyia* (*Sergentomyia*) *renauxi* (Parrot and Schwetz, 1937).

Distribution: Democratic Republic of Congo, Ethiopia, Republic of Congo.

*Sergentomyia* (*Sergentomyia*) *richardi* (Parrot and Wanson, 1946).

Distribution: Cameroon, Democratic Republic of Congo, Liberia, Togo, Uganda.

*Sergentomyia* (*Sergentomyia*) *rima* Davidson, 1987.

Distribution: Malawi, Mozambique, Namibia, South Africa, Zambia, Zimbabwe.

*Sergentomyia* (*Sergentomyia*) *rosannae* (Heisch, Guggisberg and Teesdale, 1956).

Distribution: Ethiopia, Kenya.

*Sergentomyia* (*Sergentomyia*) *ruttledgei* (Lewis and Kirk, 1946).

Distribution: Ethiopia, Sudan, Uganda.

*Sergentomyia* (*Sergentomyia*) *salisburiensis* (Abonnenc, 1967).

Distribution: Botswana, Namibia, South Africa, Zambia, Zimbabwe.

*Sergentomyia* (*Sergentomyia*) *schoutedeni* (Adler, Theodor and Parrot, 1929).

Distribution: Benin, Cameroon, Côte d'Ivoire, Democratic Republic of Congo, Ethiopia, Guinea, Kenya, Lesotho, Nigeria, Sudan, Togo, Uganda.

*Sergentomyia* (*Sergentomyia*) *schwetzi* (Adler, Theodor and Parrot, 1929).

Distribution: Algeria, Angola, Benin, Burkina Faso, Cameroon, Central African Republic, Chad, Democratic Republic of Congo, Djibouti, Egypt, Ethiopia, Gabon, Gambia, Ghana, Guinea, Côte d'Ivoire, Kenya, Liberia, Libya, Mali, Mauritania, Morocco, Mozambique, Namibia, Nigeria, Republic of Congo, Saudi Arabia, Senegal, Sierra Leone, Somalia, South Africa, Sudan, Tanzania, Togo, Tunisia, Uganda, Yemen.

*Sergentomyia* (*Sergentomyia*) *serridentata* Davidson, 1990.

Distribution: Cameroon, Uganda.

*Sergentomyia* (*Sergentomyia*) *silva* Trouillet, 1985.

Distribution: Republic of Congo.

*Sergentomyia* (*Sergentomyia*) *simillima* (Newstead, 1914).

Distribution: Benin, Burkina Faso, Cameroon, Central African Republic, Democratic Republic of Congo, Ethiopia, Ghana, Guinea, Côte d'Ivoire, Liberia, Namibia, Nigeria, Republic of Congo, Sierra, Sudan, Togo, Uganda.

*Sergentomyia* (*Sergentomyia*) *sinkiangensis* (Ting and Ho, 1962).

Distribution: China.

*Sergentomyia* (*Sergentomyia*) *sintoni* (Pringle, 1953).

Distribution: Afghanistan, Egypt, Iran, Iraq, Kuwait, Turkey, Uzbekistan.

*Sergentomyia* (*Sergentomyia*) *taizi* Lewis, 1974.

Distribution: Djibouti, Egypt, Lebanon, Saudi Arabia, Yemen.

*Sergentomyia* (*Sergentomyia*) *teteica* Artemiev, 1985.

Distribution: Mozambique.

*Sergentomyia* (*Sergentomyia*) *theodori* (Parrot, 1919).

Distribution: Afghanistan, Cyprus, Egypt, Greece, India, Iraq, Israel, Jordan, Kenya, Laos, Lebanon, Pakistan, Saudi Arabia, Syria, Turkey.

*Sergentomyia* (*Sergentomyia*) *wangi* Leng and Zhang, 1999.

Distribution: China.

*Sergentomyia* (*Sergentomyia*) *waqqsi* Artemiev, 1982.

Distribution: Iraq.

*Sergentomyia* (*Sergentomyia*) *wurtzi* (Parrot, 1938).

Distribution: Ethiopia.

*Sergentomyia* (*Sergentomyia*) *wynnae* (Watson, 1951).

Distribution: Kenya, Uganda.

*Sergentomyia* (*Sergentomyia*) *yusafi* (Sinton, 1930).

Distribution: Ethiopia, Kenya, Nigeria, Tanzania, Uganda, Yemen, Zambia.

*Sergentomyia* (*Sergentomyia*) *zumpti* (Abonnenc, 1967).

Distribution: Namibia, South Africa.

(*Sintonius*) Nitzulescu, 1931.

*Sergentomyia* (*Sintonius*) *adami* (Abonnenc, 1960).

Distribution: Central African Republic, Ethiopia, Mali.

*Sergentomyia* (*Sintonius*) *adleri* (Theodor, 1933).

Distribution: Benin, Burkina Faso, Cameroon, Central African Republic, Chad, Djibouti, Egypt, Ethiopia, Gambia, Ghana, Iran, Côte d'Ivoire, Jordan, Kenya, Niger, Nigeria, Saudi Arabia, Senegal, Sudan, Togo, Uganda, United Arab Emirates.

*Sergentomyia* (*Sintonius*) *affinis affinis* (Theodor, 1933).

Distribution: Ethiopia, Sudan.

*Sergentomyia* (*Sintonius*) *affinis vorax* (Parrot, 1948).

Distribution: Angola, Benin, Burkina Faso, Central African Republic, Chad, Ethiopia, Ghana, Guinea, Kenya, Mali, Sudan.

*Sergentomyia* (*Sintonius*) *balmicola* (Abonnenc, Adam and Bailly-Choumara, 1959).

Distribution: Cameroon.

*Sergentomyia* (*Sintonius*) *calcarata* (Parrot, 1948).

Distribution: Chad, Djibouti, Eritrea, Ethiopia, Saudi Arabia, Sudan.

*Sergentomyia* (*Sintonius*) *choumarai* (Abonnenc, 1960).

Distribution: Somalia.

*Sergentomyia* (*Sintonius*) *christophersi* (Sinton, 1927).

Distribution: Afghanistan, Algeria, Chad, Egypt, Ethiopia, Guinea, India, Iran, Jordan, Kenya, Lebanon, Libya, Morocco, Niger, Oman, Pakistan, Saudi Arabia, Yemen, Sudan, Tunisia, Uganda, Yemen.

*Sergentomyia* (*Sintonius*) *clastrieri* (Abonnenc, 1964).

Distribution: Djibouti, Guinea.

*Sergentomyia* (*Sintonius*) *clydei* (Sinton, 1928).

Distribution: Afghanistan, Algeria, Burkina Faso, Cameroon, Central African Republic Chad, Djibouti, Egypt, Eritrea, Ethiopia, Gambia, Ghana, Guinea, India, Iran, Iraq, Israel, Jordan, Kazakhstan, Kenya, Kuwait, Libya, Mali, Madagascar, Morocco, Niger, Nigeria, Oman, Pakistan, Saudi Arabia, Senegal, Seychelles, Somalia, Sudan, Tajikistan, Togo, Tunisia, Turkestan, Uganda, United Arab Emirates, Yemen.

*Sergentomyia* (*Sintonius*) *diapagai* (Abonnenc, 1962).

Distribution: Burkina Faso, Mali.

*Sergentomyia* (*Sintonius*) *eadithae* (Sinton, 1932).

Distribution: India.

*Sergentomyia* (*Sintonius*) *edentula* Pastre, 1982.

Distribution: Senegal.

*Sergentomyia* (*Sintonius*) *graingeri* (Heisch, Guggisberg and Teesdale, 1956) Distribution: Ethiopia, Kenya.

*Sergentomyia* (*Sintonius*) *herollandi* (Abonnenc, 1960).

Distribution: Mali, Senegal.

*Sergentomyia* (*Sintonius*) *hirta* (Parrot and Bouquet de Jolinière, 1945).

Distribution: Algeria.

*Sergentomyia* (*Sintonius*) *hospitii* (Sinton, 1924).

Distribution: India, Pakistan.

*Sergentomyia* (*Sintonius*) *lewisiana* Pastre, 1982.

Distribution: Ethiopia, Senegal.

*Sergentomyia* (*Sintonius*) *mbandakai* (Abonnenc, 1970).

Distribution: Democratic Republic of Congo.

*Sergentomyia* (*Sintonius*) *meilloni* (Sinton, 1932).

Distribution: Kenya, Mozambique, Namibia, South Africa, Swaziland, Zimbabwe.

*Sergentomyia* (*Sintonius*) *orissa* Kaul and Lewis, 1973.

Distribution: India.

*Sergentomyia* (*Sintonius*) *pakistanica* Artemiev and Saf'janova, 1974.

Distribution: Afghanistan, Pakistan, Turkmenistan.

*Sergentomyia* (*Sintonius*) *rogeri* Pastre, 1982.

Distribution: Senegal.

*Sergentomyia* (*Sintonius*) *sattii* Qutubuddin, 1962.

Distribution: Ethiopia, India, Sudan.

*Sergentomyia* (*Sintonius*) *sidioliensis* Pastre, 1982.

Distribution: Senegal.

*Sergentomyia* (*Sintonius*) *sirohi* Kaul, Dhanda and Modi, 1973.

Distribution: India.

*Sergentomyia* (*Sintonius*) *suberecta* (Sinton, 1932).

Distribution: Ethiopia, Kenya, Sudan.

*Sergentomyia* (*Sintonius*) *subtilis* (Parrot and Martin, 1939).

Distribution: Ethiopia.

*Sergentomyia* (*Sintonius*) *tauffliebi* (Abonnenc and Cornet, 1971).

Distribution: Côte d'Ivoire, Democratic Republic of Congo, Senegal.

*Sergentomyia* (*Sintonius*) *thomsoni thomsoni* (Theodor, 1938).

Distribution: Chad, Ethiopia, Kenya, Malawi, Mozambique.

*Sergentomyia* (*Sintonius*) *thomsoni mandarai* Krüger, Tateng and Dondji, 2019.

Distribution: Cameroon.

*Sergentomyia* (*Sintonius*) *tiberiadis* (Adler, Theodor and Lourie, 1930).

Distribution: Algeria, Afghanistan, Djibouti, Egypt, Eritrea, Ethiopia, Iran, Israel/Palestine, Lebanon, Oman, Saudi Arabia, Sudan, United Arab Emirates, Yemen.

*Sergentomyia* (*Sintonius*) *wansoni* (Parrot, 1938).

Distribution: Angola, Burkina Faso, Central African Republic, Democratic Republic of Congo, Mali, Republic of Congo, Uganda.

(*Trouilletomyia*) Depaquit and Léger, 2014.

*Sergentomyia* (*Trouilletomyia*) *boironis* Randrianambinintsoa and Depaquit, 2014.

Distribution: Madagascar.

*Sergentomyia* (*Trouilletomyia*) *huberti* Depaquit, Léger and Robert, 2002.

Distribution: Madagascar.

(*Vattieromyia*) Depaquit, Léger and Robert, 2008.

*Sergentomyia* (*Vattieromyia*) *anka* Depaquit, Léger and Robert, 2008.

Distribution: Madagascar.

*Sergentomyia* (*Vattieromyia*) *namo* Depaquit, Léger and Robert, 2008.

Distribution: Madagascar.

*Sergentomyia* (*Vattieromyia*) *pessoni* Depaquit, Randrianambinintsoa and Léger, 2012.

Distribution: Union of the Comoros.

*Sergentomyia* (*Vattieromyia*) *sclerosiphon* Depaquit, Léger and Robert, 2008.

Distribution: Madagascar.

*Incertae sedis*

*Sergentomyia angustipennis* (de Meijere, 1909).

Distribution: Indonesia.

*Sergentomyia anodontis* (Quate and Faichild, 1961).

Distribution: Cambodia, Malaysia Thailand.

*Sergentomyia bailyi* (Sinton, 1931).

Distribution: Cambodia, Cameroon, China, India, Laos, Pakistan, Sri Lanka, Thailand, Vietnam.

*Sergentomyia bandjara* Lewis, 1987.

Distribution: Indonesia, Malaysia.

*Sergentomyia bernardae* Trouillet, 1982.

Distribution: Republic of Congo.

*Sergentomyia brachycornuta* (Fairchild, 1952).

Distribution: Indonesia.

*Sergentomyia brevicaulis* (Quate, 1962).

Distribution: Laos, Thailand, Vietnam.

*Sergentomyia brunhesi* Léger, Randrianambinintsoa and Depaquit, 2020.

Distribution: Madagascar.

*Sergentomyia bukidnonis* (Quate, 1965).

Distribution: Philippines.

*Sergentomyia cheongi* Lewis and Jeffery, 1978.

Distribution: Malaysia.

*Sergentomyia cherukara* (Kaul, 1933).

Distribution: India.

*Sergentomyia cidaria* (Quate and Quate, 1967).

Distribution: Papua-New Guinea.

*Sergentomyia cunicula* Davidson, 1979.

Distribution: Namibia.

*Sergentomyia curtata* (Quate and Quate, 1967).

Distribution: Indonesia.

*Sergentomyia dapsilidentes* (Quate, 1965).

Distribution: Philippines.

*Sergentomyia delfinadoae* (Quate, 1965).

Distribution: Philippines.

*Sergentomyia dentacea* (Quate, 1965).

Distribution: Philippines.

*Sergentomyia displicata* (Quate and Fairchild, 1961).

Distribution: Malaysia.

*Sergentomyia dvoraki* Randrianambinintsoa, Vongphayloth and Depaquit, 2024.

Distribution: Laos.

*Sergentomyia exastis* (Quate, 1965).

Distribution: Philippines.

*Sergentomyia fanglianensis* (Leng, 1964).

Distribution: China.

*Sergentomyia fergusoni* (Fairchild, 1952).

Distribution: Indonesia.

*Sergentomyia franciscana* (Quate, 1965).

Distribution: Philippines.

*Sergentomyia fukienensis* (Tang, 1959).

Distribution: China.

*Sergentomyia gibsoni* Lewis and Dyce, 1989.

Distribution: Australia.

*Sergentomyia hassani* Lewis, 1978.

Distribution: Malaysia.

*Sergentomyia hiverna* (Raynal and Gaschen, 1935).

Distribution: Laos, Thailand, Vietnam.

*Sergentomyia horridula* Vattier-Bernard and Trouillet, 1982.

Distribution: Republic of Congo.

*Sergentomyia hunti* (Lewis and Kirk, 1946).

Distribution: Central African Republic, Sudan.

*Sergentomyia imitor* (Quate, 1965).

Distribution: Philippines.

*Sergentomyia iriomotensis* Sanjova and Miyag, 2022.

Distribution: Japan.

*Sergentomyia jamesi* Lewis, 1978.

Distribution: Thailand, Sri Lanka.

*Sergentomyia kachekensis* (Yao and Wu, 1938).

Distribution: China.

*Sergentomyia knudseni* Lewis and Jeffery, 1978.

Distribution: Malaysia.

*Sergentomyia koraputa* Lewis and Kaul, 1987.

Distribution: India.

*Sergentomyia lanzhouensis* Xiang, Jun and Zuo, Chai, 2006.

Distribution: China.

*Sergentomyia losarca* (Quate, 1965).

Distribution: Philippines.

*Sergentomyia maai* (Quate and Fairchild, 1961).

Distribution: Malaysia.

*Sergentomyia mahadevani* Lewis, 1978.

Distribution: Thailand.

*Sergentomyia maiae* Depaquit, Renaux Torres and Siriyasatien, 2023.

Distribution: Thailand.

*Sergentomyia majungaensis* Depaquit, Léger and Robert, 2007.

Distribution: Madagascar.

*Sergentomyia marolii* Vongphayloth, Randrianambinintsoa and Depaquit, 2024.

Distribution: Laos.

*Sergentomyia metzi* Davidson, 1979.

Distribution: Namibia, South Africa.

*Sergentomyia montana* (Sinton, 1924).

Distribution: India, Pakistan, Nepal.

*Sergentomyia morini* (Raynal and Gaschen, 1935).

Distribution: Vietnam.

*Sergentomyia musai* Lewis, 1978.

Distribution: Malaysia.

*Sergentomyia neras* (Quate, 1965).

Distribution: Philippines.

*Sergentomyia nicnic* (Banks, 1919).

Distribution: Philippines.

*Sergentomyia noenforensis* (Fairchild, 1952).

Distribution: Indonesia.

*Sergentomyia pachystoma* (Quate and Fairchild, 1961).

Distribution: Malaysia.

*Sergentomyia phasukae* Curler, 2011.

Distribution: Thailand.

*Sergentomyia pooi* (Yao and Wu, 1941).

Distribution: China.

*Sergentomyia pugifera* Lewis and Dyce, 1989.

Distribution: Australia.

*Sergentomyia quinta* (Faichild, 1952).

Distribution: Indonesia.

*Sergentomyia raynali* Depaquit, Siriyasatien and Polseela, 2019.

Distribution: Laos, Thailand.

*Sergentomyia reidi* (Lewis, 1957).

Distribution: Malaysia.

*Sergentomyia roberti* Vattier-Bernard and Trouillet, 1981.

Distribution: Democratic Republic of Congo, Republic of Congo.

*Sergentomyia shettyi* Ilango, 1994.

Distribution: India.

*Sergentomyia sibylas* (Quate and Quate, 1967).

Distribution: Indonesia.

*Sergentomyia smithi* (Mitra and Roy, 1952).

Distribution: India.

*Sergentomyia standifasti* Lewis and Dyce, 1989.

Distribution: Australia.

*Sergentomyia tangi* Xiong and Chai, 2006.

Distribution: China.

*Sergentomyia tracheola* Xiong and Jin and Chai, 2006.

Distribution: China.

*Sergentomyia traubi* (Lewis, 1957).

Distribution: Malaysia.

*Sergentomyia turfanensis* Hsiung, Guan and Jin, 1981.

Distribution: China.

*Sergentomyia vanella* (Quate and Quate 1967).

Distribution: Indonesia.

*Sergentomyia villosa* Davidson, 1979.

Distribution: Namibia, South Africa, Zimbabwe.

*Sergentomyia vistellei* Depaquit, Randrianambisintsoa and Léger, 2020.

Distribution: Madagascar.

*Sergentomyia vulpes* Davidson, 1979.

Distribution: Namibia, South Africa.

*Sergentomyia welwitschii* Davidson, 1979.

Distribution: Namibia, South Africa.

*Sergentomyia yoshimotoi* (Quate, 1965).

Distribution: Philippines.

*Sergentomyia yunnanenis* He and Leng, 1991.

Distribution: China.

*Sergentomyia zielkei* Seccombe, Ready and Huddleston, 1993.

Distribution: South Africa.

Not assigned in any tribe.

†*Libanophlebotomites* Azar, Maalouf and Maksoud, 2022 (1 species).

†*Libanophlebotomites ramyi* Azar, Maalouf and Maksoud, 2022.

Distribution: Lebanon.

†*Libanophlebotomus* Azar, Nel, Solignac, Paicheler and Bouchet, 1999 (1 species).

†*Libanophlebotomus lutfallahi* Azar, Nel, Solignac, Paicheler and Bouchet, 1999 Distribution: Lebanon.

†*Mesophlebotomites* Azar, Nel, Solignac, Paicheler and Bouchet, 1999 (1 species).

†*Mesophlebotomites hennigi* Azar, Nel, Solignac, Paicheler and Bouchet, 1999.

Distribution: Lebanon.

†*Paleomyi*a Poinar, 2004 (1 species).

†*Paleomyi*a *burmitis* Poinar, 2004.

Distribution: Myanmar.

† *Phlebotoiella* Kraemer and Wagner, 2009(1 species).

† *Phlebotoiella eoindianensis* Kraemer and Wagner, 2009.

Distribution: India.

## Discussion

The current checklist is based on various general articles and reviews cited in the material and methods, supplemented by more recent original articles. We also provide some unpublished data. Species that have not been formally described or named following ICZN standards are noted for reference outside the checklist and are not included in the published dataset in GBIF. Furthermore, clarifications have been made regarding the taxonomic position of certain doubtful species. Following Shimabukuro et al. [20] 14 species names that fall under articles 17.2 and 23.8 of ICZN were excluded from the list: *Dampfomyia* sp. of Suchitepequez (Young and Duncan, 1994), *Evandromyia* (*Aldamyia*) sp. of Baduel (Floch and Abonnenc, 1945), *Lutzomyia* (*Helcocyrtomyia*) sp. of Pichinde Young, 1979, *Micropygomyia* (*Sauromyia*) sp. 2 of Araracuara (Morales and Minter, 1981), *Pintomyia* sp. of Anchicaya (Young, 1979), *Pressatia* #1 Mangabeira, 1942, *Psychodopygus* sp. of Trés Esquinas (Young, 1979), *Sergentomyia* (*Neophlebotomus*) Besout sp. Lewis, 1978, *Sergentomyia* (*Neophlebotomus*) Rabok sp. Lewis, 1978, *Sergentomyia* (*Neophlebotomus*) Sepilok sp., Lewis, 1978, *Sergentomyia* (*Parrotomyia*) sp. A Kaul Dhanda and Modi, 1973, *Sergentomyia* (*Parrotomyia*) sp. B Kaul Dhanda and Modi, 1973, *Sergentomyia* (Ungrouped) Okinawa sp. Lien, 1975, *Trichophoromyia* sp. 1 of Araracuara (Morales and Minter, 1981).

After 2017, 16 sand fly species were described in South America. Here, the classification of *Lutzomyia ignacioi* and *Lutzomyia ponsi* has been updated, removing them from the genus *Psathyromyia* and adding them to the genus *Lutzomyia* [41]. Shimabukuro et al. [20] included *Lu.* (*Hel.*) *sanguinaria* and *Lu.* (*Hel.*) *duncanae* in Peru, but according to Roberto Fernández (personal communication), it is dubious and was excluded from the present study. *Bichromomyia olmeca* and *Bichromomyia bicolor* were considered as species rather than subspecies [46]. 

The genus *Idiophlebotomus*, which has been understudied due to its lack of involvement in the transmission of *Leishmania* or arboviruses, presents some uncertainties for some records outside their type localities. *Idiophlebotomus longiforceps* has been described with limited details from China and its records in Southeast Asia may not be valid. Similarly, the record of *Id. teshi* in Thailand, a species originally described from Nepal, is likely questionable.

In the genus *Phlebotomus*, three species have been omitted from the subgenus *Anaphlebotomus* described from India [19, 47, 48]: *Ph. chiyankiensis* Singh, Phillips-Singh and Ipe, 2009, *Ph. palaumensis* Singh, Phillips-Singh and Ipe, 2007 and *Ph. sanctijohani* Ipe and Singh, 1994. These species share evident characters suggesting including them within the genus *Sergentomyia*, but the descriptions lack sufficient details to determine whether they are junior synonyms of *Sergentomyia* species already described. In the subgenus *Euphlebotomus*, *Ph. argentipes* is the main vector of *Leishmania donovani*, especially in the Indian subcontinent. The taxonomic status of *Ph. argentipes* as a species complex has been debated for decades, with conflicting interpretations regarding its composition as a complex of two or three distinct members. Illango et al. [49] described the *Ph. argentipes* species complex as comprising three distinct species complex members (*Ph. annandalei*, *Ph. argentipes* s.s., and *Ph. glaucus*) based solely on morphometric analyses. However, recent studies employing advanced molecular techniques such as COI and Cytb gene amplification [50–52] have only characterized these as complex members without identifying them as separate species. Instead, these studies have referred to the members as “group A,” “group B,” or “morphospecies A and B,” based on molecular analysis. Given the absence of molecular evidence and the reliance solely on morphological data, it is far fetched to corroborate *Ph. annandalei* and *Ph. glaucus* as valid species in the modern molecular era. Therefore, the name *Ph. argentipes* used in this work includes all populations and possible species mentioned above. The populations reported from Southeast Asia as *Ph. argentipes* may represent a distinct species [53]. Moreover, the validity of the description of Chinese species *Ph. kiangsuensis* is questionable, and records of this species in Southeast Asia likely refer to another distinct species, yet to be described. Within the subgenus *Larroussius*, the species *Ph. major* holds its name to a species complex including *Ph. neglectus*, *Ph. syriacus*, *Ph. wenyoni,*, and *Ph. major s.s.* The records of the latter species in Southeastern Asia appear erroneous, while those in Europe or the Near East refer to *Ph. neglectus*. The boundaries between species included in the *perfiliewi* group *(Ph. galilaeus*, *Ph. perfiliewi s.s.* and *Ph. transcaucasicus*) are not clearly defined [54], and the record of *Ph. transcaucasicus* in Israel/Palestine may refer to *Ph. galilaeus*. Old records of *Ph. perniciosus* in Bulgaria or the southern Balkans likely correspond to *Ph. tobbi*. Finally, the inclusion of *Ph. somaliensis* in the subgenus *Larroussius* is debatable. Known only from the female and poorly documented, its spermatheca seems slightly different from the typical morphological criteria described for this subgenus. The distribution of species in the subgenus *Lewisius* deserves to be clarified. The type species of this recently described subgenus [22] is *Ph. betisi*. This species was formerly considered as *Larroussius*. The records of this species outside Malaysia should be re-evaluated as they may refer to *Ph. breyi* or another species. In the subgenus *Paraphlebotomus*, the subtle distinction between *Ph. sergenti* and *Ph. similis* has probably led to erroneous records. Subject to updating of the data, the presence of *Ph. similis* in the Balkans or Romania appears questionable, a region in which only *Ph. sergenti* seems to be present. The Indian species *Ph. sikandraensis* belongs to the subgenus *Synphlebotomus* and not *Euphlebotomus* as incorrectly indicated in the summary of the original description [55]. 

In the genus *Grassomyia*, given the endemism in Madagascar, it is unlikely that *Gr. squamipleuris* should be recorded on this island.

Regarding the genus *Sergentomyia*, *Se. barraudi* and *Se. iyengari* have been originally described from India and widely reported across Southeast Asia. However, they most likely constitute two species complexes requiring further clarification, including the study of type specimens. Therefore, their taxonomy and geographical distributions are questionable and should be used with caution. For* Se. iyengari s.l.*, several populations have been described as subspecies, then considered as species (*Se. khawi*,* Se. hivernus*,* Se. iyengari sensu* Raynal now described as* Se. dvoraki* [28]) or synonymized by several authors, i.e.* Se. hainanensis*,* Se. malayensis*, and* Se. taiwanensis*. There are currently no arguments to decide the status of these populations. In the subgenus *Parrotomyia*, the records of *Se. grekovi* in Romania and *Se. rudnicki* in Sri Lanka need to be validated. We have considered *Se. lewisi* as a junior synonym of *Se. palestinensis*. In the subgenus *Rondanomyia*, despite several records across various countries, the identity of *Se. perturbans* remains doubtful due to its confused history, particularly outside Indonesia. Additionally, the records of *Se. linearis* in Malaysia and *Se. malayae* in Sri Lanka need validation. In the subgenus *Sergentomyia*, the species limits within the group *bedfordi* remain challenging to define [56].

Regarding the fossil species, the wing shape of *Ph. pungens* appears too rounded at the tip to be classified in the genus *Phlebotomus*. Additionally, the very rounded wing of *Ph. vetus* indicates it probably belongs to another genus. Furthermore, the position of the four spines on the gonostyle does not correspond to any known subgenus of *Phlebotomus*.

## Conclusions

This review is based on several publications of Phlebotominae around the world since the first species’ description. Also, unpublished personal collection data from each author allowed this review to be the most complete list of sand flies ever published. However, one limitation was the inability to verify the records for each country of the articles reviewed. It emphasizes the importance of depositing slides in collections to future research. This list is now part of GBIF, and it can be updated in real time when a new species is described or registered globally. Moreover, the problem of changing borders or country names raises many ambiguities such as the use of “regions.” The example of certain Old World genera, especially some African species, with the evolution of names (i.e., Congo referring to the Democratic Republic of Congo (formerly Congo Kinshasa or Congo Belge) or the Republic of Congo (formerly Congo-Brazzaville)) can lead to confusion as well as the creation of new countries (i.e., former republics of the USSR, Eritrea, or South Sudan). Likewise, the use of determination keys for regions, for example, “Ethiopian Region” or “Guinean Savanna Province”, without the species being necessarily present in the countries giving their names to the concerned region (i.e., Ethiopia and Guinea) can lead to subsequent errors concerning localities. Only articles with mention of the exact country (and, if possible, a precise locality) have been included in the present study.

## Data Availability

The dataset for this study is publicly available in the “Sistema de Informação sobre a Biodiversidade Brasileira (SiBBr)” and the Global Biodiversity Facility Information (GBIF) (10.15468/54hqx7).
